# Mechanisms and Thermochemistry of Reactions of SiO and Si_2_O_2_ with OH and H_2_O

**DOI:** 10.1021/acs.jpca.3c00862

**Published:** 2023-05-02

**Authors:** Stefan Andersson

**Affiliations:** Department of Metal Production and Processing, SINTEF, P.O. Box 4760 Torgarden, 7465 Trondheim, Norway

## Abstract

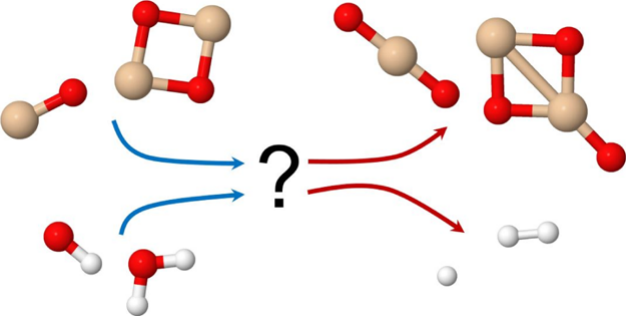

This paper reports on computational studies of gas-phase reactions of SiO and Si_2_O_2_. The oxidation of SiO can initiate efficient formation of silica or silicate dust particles in a wide range of environments. Both OH radicals and H_2_O molecules are often present in these environments, and their reactions with SiO and the smallest SiO cluster, Si_2_O_2_, affect the efficiency of eventual dust formation. Density functional theory calculations on these reactions, benchmarked against accurate coupled cluster calculations, indicate that the Si_2_O_2_ + OH reaction should be faster than SiO + OH. The reaction SiO + H_2_O → SiO_2_ + H_2_ is both endothermic and has high activation energies to reaction. Instead, the formation of molecular complexes is efficient. The reaction of Si_2_O_2_ with H_2_O, which has been suggested as efficient for producing Si_2_O_3_, might not be as efficient as previously thought. If the H_2_O molecules dissociate to form OH radicals, oxidation of SiO and Si_2_O_2_ could be accelerated instead.

## Introduction

Molecular SiO has been observed in interstellar and circumstellar space and is believed to be a key component for the formation of silicate particles, which make up a significant part of observed interstellar dust.^1−16^ SiO molecules have also been predicted to exist in the upper atmosphere (from meteoric ablation and subsequent reactions)^17^ and have been found in combustion of silicon compounds^18−24^ and in industrial silicon production processes.^25−31^ SiO can react with oxygen-bearing species, such as OH and O_2_ to form SiO_2_.^16,32^

Molecular SiO_2_ will condense to SiO_2_ clusters and eventually silica particles under normal conditions.^18,22,31^ It has been suggested that SiO also could condense directly to form clusters, the simplest being Si_2_O_2_, and through a series of reactions with different species form silica or silicate dust.^10−14^ Experimental data on details of the reactions of SiO oxidation are scarce.^32^ Quantum chemical and rate theory calculations therefore become important to quantify the reaction mechanisms^10−13,32−36^

Combustion of silicon compounds^18^ such as silane,^19−22^ SiH_4_, silicon tetrachloride,^23^ SiCl_4_, and hexamethyldisiloxane (HMDS),^24^ C_6_H_18_Si_2_O, lead to the formation of *fumed* or *pyrogenic silica* consisting of nano- to micrometer sized particles of amorphous SiO_2_. Fumed silica has found widespread use in industry. Proposed reaction schemes involve the reactions of SiO with O_2_ and OH as important intermediate steps.

In silicon and ferrosilicon smelting plants, liquid silicon or silicon alloy is produced from a mixture of quartz and carbonaceous materials (coke, coal, or wood) that is heated in an electric arc furnace.^25−31^ Gaseous CO and SiO are released in the process and these are subsequently oxidized in contact with air in the hot environment (about 2000 K). Reactions with oxygen-containing species yield CO_2_ and silica dust as products. However, the exact mechanism behind the silica dust formation remains unclear. There is a correlation between silica dust formation and NO_*x*_ production,^26−30^ the details of which are only partly understood. Silica dust is also formed during tapping and refining of silicon.^31,37^ Emission of ultrafine dust into a workplace atmosphere may cause adverse health effects, potentially both asthma and chronic obstructive pulmonary disease.^37^ The additional NO_*x*_ production means that the dust formation process has an additional impact as a potential health and environmental hazard, which should therefore preferably be minimized. Efficient filters and ventilation are essential to protect workers from exposure to dust.

Silicon enters the upper atmosphere through the ablation of silicate-containing interplanetary dust particles.^17^ This results in gas-phase Si and SiO that can react further with oxygen species, like O_3_ and OH, to eventually form SiO_2_.

The reactions
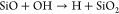
1and
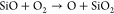
2have been studied both experimentally and theoretically. The only reported experimentally determined rate constants for the SiO + OH reaction consists of one measured value at 293 K.^32^ There are a few computational studies on the reaction. Zachariah and Tsang^33^ calculated a temperature-dependent expression for the rate constant based on electronic structure calculations (Hartree–Fock structures and frequencies and MP4 energies) and RRKM theory. At 293 K, these rate constants differ by an order of magnitude, the experimental value being (5.9 ± 1.4) × 10^–12^ cm^3^ s^–1^ with a theoretical value of 3.1 × 10^–13^ cm^3^ s^–1^. Apart from the study mentioned above, the work on this reaction was reported by Gómez Martín et al.^32^ and Plane,^11^ where they used electronic structure calculations and master equation rate calculations to interpret the room-temperature experiments and to extrapolate to lower and higher temperatures. They optimized stationary points on the HOSiO potential energy surface (PES) using B3LYP/6-311+G(2d,p) and subsequently used these structures to calculate the thermochemistry with the CBS-Q method. Based on these data, RRKM/master equation calculations were made to estimate the pressure dependence of the rate constant at 296 K. Both studies indicate that the rate of reaction (1) has a clear pressure dependence and is dominated by the formation of a stabilized HOSiO intermediate at pressures above approximately 0.1 atm. Hao et al.^34^ calculated stationary points on the HOSiO PES for the study of reaction (1) using coupled cluster [CCSD(T)] calculations with the cc-pV(Q+d)Z basis set, thus providing higher accuracy molecular structures and energies than the previous studies. In addition, there are a number of estimated rate constants that have been used in both combustion^19,23^ and astrochemistry modeling.^1,2,4−6^ Even if one removes both the highest and lowest estimates, there is still a spread of values of 4 orders of magnitude at room temperature and 2 orders of magnitude at 2000 K and it is unclear which type of temperature dependence the rate follows. At a typical interstellar temperature of 10 K, the calculated rate by Zachariah and Tsang^33^ is vanishingly small, whereas values typically used in astrochemistry modeling would lead to appreciable reaction. Obviously, there is need for further study on this reaction. The reaction of Si_2_O_2_ with OH has to the best of our knowledge not been studied previously.

Gómez Martín et al.^32^ also attempted to measure the rate constant of reaction 2 (SiO + O_2_) at 223 and 293 K but could only establish an upper limit of 4.5 × 10^–15^ cm^3^ s^–1^. Supporting ab initio calculations indicated that this reaction is endothermic and involves substantial barriers, which would make it very slow at room temperature and it is probably only important in combustion systems.

Information on the energetics of the reaction of SiO with H_2_O can be found in several papers reporting on electronic structure calculations on parts of the PES.^32,33,35,36^ To the best of our knowledge, the only computational study of any barrier to the Si_2_O_2_ + H_2_O reaction was made by Zachariah and Tsang,^33^ and they only studied one transition state, leading to the formation of one specific type of molecular complex. There have been no reported attempts to study reaction pathways to the formation of Si_2_O_3_ + H_2_.

Based on their potential importance for dust formation in a wide range of environments, we decided to study the reactions between SiO and Si_2_O_2_ with OH radicals and H_2_O molecules, respectively.

## Computational Details

Quantum chemistry techniques have reached a level of maturity, such that the structures, spectroscopy, and thermochemistry of small molecules can be calculated to an accuracy rivaling experiments.^38,39^ We carried out calculations using the CCSD(T) (coupled cluster with single and double excitations and a perturbative treatment of triple excitations) method, since this is considered to be the “gold standard” of quantum chemistry, allowing to calculate energy differences to “chemical accuracy”, that is, with an error below 1 kcal/mol. In short, this involves calculating the correlation of electrons as an improvement on the mean-field Hartree–Fock electronic wavefunction that is used as a reference state. For calculating the enthalpies of formation of some key molecular species, we followed a procedure where first the molecular geometry was optimized using CCSD(T) with the basis set aug-cc-pVQZ^40^ for H and O and aug-cc-pV(Q+d)Z for Si^41^ using the commonly employed frozen-core approximation. For open-shell species, such as OH, unrestricted Hartree–Fock (UHF) wavefunctions were used as reference states for the CCSD(T) calculations. Subsequently, harmonic vibrational frequencies were calculated using the same method. Using the optimized geometries, frozen-core calculations with CCSD(T) and the larger aug-cc-pv5Z, aug-cc-pV6Z,^42^ aug-cc-pV(5+d)Z, and aug-cc-pV(6+d)Z^41^ basis sets were carried out in order to approach the complete basis set (CBS) limit. The calculated energy was further corrected for core-valence (CV) electron correlation, where not only valence but also outer core electrons (1s for H and O and 2s and 2p for Si) were correlated in the CCSD(T) calculations (using the cc-pwCVTZ, cc-pwCVQZ, and cc-pwCV5Z basis sets^43^). Both the frozen-core and CV calculations were extrapolated to the CBS limit using the extrapolation formula^44,45^*E*(CBS) = *E*(*l*_max_) + *A*/(*l*_max_ + 1/2)^4^. Finally, a first-order relativistic correction was added by employing all-electron CCSD(T) with an uncontracted cc-pVTZ^46,47^ basis set and the DPT2 direct perturbation method.^48,49^ All coupled cluster calculations were performed using the CFOUR software package.^50,51^

The enthalpy of formation and standard entropy were calculated by standard statistical thermodynamics equations,^52^ employing calculated vibrational frequencies, rigid-rotor rotational constants calculated from the optimized geometries, and experimental data on electronic fine-structure states.^53^ The standard state of Si at 298 K is the solid state, but for practical reasons, the Si atom was used as reference species in the CCSD(T) calculations. We therefore employed the most accurate estimate of the enthalpy of formation of the Si atom available,^54^ to adjust the enthalpy of formation to the correct reference value. A conservative estimate of the accuracy of the enthalpy of formation is obtained by summing up the expected uncertainties in the various contributions to total energy. The neglect of higher-order electron correlation in the CCSD(T) method should give at most 1 kcal/mol (4.184 kJ/mol) uncertainty. In the case of H and H_2_, the uncertainty has been taken to be that of sub-chemical accuracy (0.1 kcal/mol), since no higher-order excitations are possible in a two-electron system. The error bar on the enthalpy of formation of the Si atom is 0.2 kcal/mol.^54^

For optimizing molecular structures of species involved in the reactions under study, we have tested three different density functionals: B3LYP,^55,56^ M06-2X, and M06^57,58^ as well as two different basis sets: 6-31+G(d,p) and maug-cc-pVTZ [maug-cc-pV(T+d)Z for Si].^59^ All density functional theory (DFT) calculations were performed using the NWChem program package.^60^

In order to provide benchmark energetics for the reactions, we performed single-point CCSD(T)/aug-cc-V(5+d)Z calculations on molecular geometries optimized using M06/maug-cc-pV(T+d)Z. These calculations were run using CFOUR.

## Results and Discussion

### Benchmark Energetics

Results for calculating the standard enthalpies of formation of species relevant to our study are presented in Table 1. The benchmark CCSD(T)/CBS results for SiO, OH, H_2_O, and H agree well with literature values. For the gas-phase SiO_2_ molecule, there is much larger discrepancy with literature, but the accuracy of our calculations suggests that the literature data should be revised. Of the density functionals, M06 shows close agreement with CCSD(T), whereas the M06-2X and B3LYP results clearly deviate more from CCSD(T). In the case of Si_2_O_2_, there is a large discrepancy with the literature value, but the experimental error bars are very large and also cover the CCSD(T) value with M06 differing from CCSD(T) by only 0.5 kJ/mol. M06-2X differs by 8 kJ/mol from CCSD(T), whereas the deviation for B3LYP is as much as 80 kJ/mol. Calculated CCSD(T) and M06 geometries and vibrational frequencies of the species in Table 1 are compared to the experimental data in Table S1 in the Supporting Information.

**Table 1:** Calculated and Tabulated Standard Enthalpies of Formation^a^

	Δ*H*_f,298_° (kJ/mol)	Δ*H*_f,298_° (kJ/mol)	Δ*H*_f,298_° (kJ/mol)	Δ*H*_f,298_° (kJ/mol)	Δ*H*_f,298_° (kJ/mol)	Δ*H*_f,298_° (kJ/mol)	Δ*H*_f,298_° (kJ/mol)
species	CCSD(T)^b^	M06^b^^,^^c^	M06-2X^b^	B3LYP^b^	prev. calc	JANAF^d^	other
SiO	–100.47 ± 4.8	–97.8	–92.0	–72.4	–111.7 ± 15.9^e^	–100.42 ± 8.4	
					–101.9^f^		
					–94.6^g^		
SiO_2_	–286.12 ± 4.8	–278.8	–261.9	–235.5	–282.0^e^	–305.43 ± 33.5	–322.07 ± 10^h^
					–288.0^f^		
					–277.0^g^		
Si_2_O_2_	–430.49 ± 5.4	–431.4	–438.7	–349.8	–385.8 ± 45.2^e^		–465.3 ± 42^i^
OH	36.40 ± 4.2	36.2	37.9	40.3		38.987 ± 1.21	37.36 ± 0.13^j^
H_2_O	–242.34 ± 4.2	–242.4	–237.1	–224.7		–241.826 ± 0.042	–241.81 ± 0.03^j^
H	217.81 ± 0.42	214.3	214.8	219.3		217.999 ± 0.006	217.998 ± 0.006^k^

a The CCSD(T) data are calculated in the complete basis set limit, whereas DFT (M06, M06-2X, and B3LYP) data use the maug-cc-pVTZ basis set.

b This work.

c Reference (61).

d Reference (62).

e Reference (63).

f Reference (33).

g Reference (64).

h Reference (65).

i Reference (66).

j Reference (67).

k Reference (68).

In order to make a proper choice of density functional for reliable studies of the reaction energetics of Si–O–H systems, we tested the B3LYP, M06-2X, and M06 density functionals toward CCSD(T) benchmark calculations for stationary points on the PESs of the reactions involving SiO. Single-point CCSD(T)/aug-cc-pV(5+d)Z calculations are performed using the M06/maug-cc-pV(T+d)Z molecular geometries. To minimize the risk that this creates a pronounced bias toward the M06 functional, we also include results for the reaction energies from CCSD(T)/aug-cc-pV(5+d)Z and CCSD(T)/aug-cc-pV(6+d)Z single-point calculations using CCSD(T)/aug-cc-pV(Q+d)Z geometries. In Table 2 we show a comparison between stationary points for the SiO + OH reaction (discussed in a subsection below). Mean signed errors (MSE) and mean unsigned errors (MUE) are given for the density functionals, calculated from the difference in energy to the CCSD(T) results. As can be seen, the M06/maug-cc-pV(T+d)Z results agree extraordinarily well with the benchmark energetics with mean errors below 2 kJ/mol. M06-2X and B3LYP are significantly less accurate, albeit not in error by more than 8 kJ/mol. It is also interesting to note the importance of using a sufficiently large basis set. Using a double-zeta (DZ) basis set, 6-31+G(d,p), together with M06 gives the poorest performance of the four sets of DFT calculations. Another interesting observation is that for the analogous reaction with carbon instead of silicon, that is, CO + OH, we found in a previous study that M06-2X gave remarkably accurate energetics for the PES and M06 gave significantly less accurate results.^69^ Great care is clearly needed for making the correct choice of density functional for studying a system with a new chemical composition.

**Table 2:** Comparison of DFT Energies to Benchmark CCSD(T) Energies for Stationary Points (Minima and Transition States) on the PES for the SiO + OH Reaction^a^

	M06	M06	M06-2X	B3LYP	CCSD(T)	CCSD(T)	CCSD(T)
	DZ^b^	TZ^c^	TZ^c^	TZ^c^	5Z//M06^d^	5Z//QZ^e^	6Z//QZ^f^
SiO + OH	0.0	0.0	0.0	0.0	0.0	0.0	0.0
H + SiO_2_	37.2	7.1	20.0	28.1	7.8	7.0	5.5
OH–SiO	–35.0	–31.4	–31.9	–28.3	–29.0		
TS1	–28.4	–25.9	–27.8	–20.8	–20.2		
*cis*-HOSiO	–249.0	–268.5	–276.8	–257.1	–267.8		
TS2	–232.7	–253.1	–260.6	–242.3	–251.7		
*trans*-HOSiO	–245.1	–265.8	–272.0	–255.6	–265.1		
TS3	–32.6	–61.5	–54.7	–59.9	–62.9		
HSiO_2_	–104.2	–126.9	–111.6	–120.3	n.a^g^		
TS4	–61.2	–84.9	–92.0	–72.6	–83.6		
TS5	54.4	27.4	n.a^g^	32.3	27.6		
MSE	15.7	–1.4	–2.9	8.0			
MUE	19.2	1.8	8.0	8.1			

a Electronic energies relative to SiO + OH are given in kJ/mol. See text for details.

b 6-31+G(d,p).

c maug-cc-pV(T+d)Z.

d CCSD(T)/aug-cc-pV(5+d)Z//M06/maug-cc-pV(T+d)Z.

e aug-cc-pV(5+d)Z//aug-cc-pV(Q+d)Z.

f aug-cc-pV(6+d)Z//aug-cc-pV(Q+d)Z.

g No converged result.

In Table 3 we present a similar type of comparison as above, but now for the SiO + H_2_O reaction. For this case, the M06/maug-cc-pV(T+d)Z and B3LYP/maug-cc-pV(T+d)Z results are roughly equally accurate with MUEs of 6 kJ/mol. Again, M06-2X is less accurate, and M06/6-31+G(d,p) shows poor performance also in this case.

**Table 3:** Comparison of DFT Energies to Benchmark CCSD(T) Energies for Stationary Points (Minima and Transition States) on the PES for the SiO + H_2_O Reaction^a^

	M06	M06	M06-2X	B3LYP	CCSD(T)	CCSD(T)	CCSD(T)
	DZ^b^	TZ^c^	TZ^c^	TZ^c^	5Z//M06^d^	5Z//QZ^e^	6Z//QZ^f^
SiO + H_2_O	0.0	0.0	0.0	0.0	0.0	0.0	0.0
H2 + SiO_2_	103.3	76.4	86.1	78.3	74.5	73.7	72.5
H_2_O–SiO	–35.8	–31.9	–33.0	–22.6	–25.4		
TS9	171.2	159.7	161.6	161.0	165.6		
TS10	7.5	8.0	–1.6	17.0	14.3		
Si(OH)_2_–S	–162.2	–173.3	–182.6	–158.6	–166.3		
TS11	–131.9	–146.1	–155.5	–132.0	–138.7		
Si(OH)_2_–M	–151.1	–165.9	–175.2	–152.2	–160.6		
TS12	–137.3	–149.0	–158.1	–134.2	–140.4		
Si(OH)_2_–C	–151.2	–163.9	–172.3	–149.1	–155.8		
TS13	121.5	95.9	98.6	99.3	95.9		
TS14	125.1	98.0	100.8	100.3	97.7		
*trans*-HSiOOH	–117.8	–141.5	–135.5	–123.5	–134.3		
TS15	–88.4	–113.9	–108.8	–96.5	–106.0		
*cis*-HSiOOH	–102.3	–128.1	–122.3	–110.2	–121.1		
TS16	67.1	38.5	45.0	56.8	48.5		
TS17	170.8	132.9	145.9	142.2	137.3		
TS18	313.4	292.1	308.8	278.1	281.0		
MSE	13.9	–4.6	–3.8	5.2			
MUE	15.9	6.2	10.1	6.1			

a Electronic energies relative to SiO + H_2_O are given in kJ/mol. See text for details.

b 6-31+G(d,p).

c maug-cc-pV(T+d)Z.

d CCSD(T)/aug-cc-pV(5+d)Z//M06/maug-cc-pV(T+d)Z.

e aug-cc-pV(5+d)Z//aug-cc-pV(Q+d)Z.

f aug-cc-pV(6+d)Z//aug-cc-pV(Q+d)Z.

The final relevant case for evaluating the accuracy of the density functionals is the formation of small Si_2_O_*x*_ species (*x* = 2, 3, 4) from SiO and SiO_2_ (Table 4). In this case, the M06/maug-cc-pV(T+d)Z are more accurate than the other density functionals by tens of kJ/mol. Based on these results, we chose to use M06/maug-cc-pV(T+d)Z for the evaluation of reaction mechanisms and energetics of the reactions under study. This also seems to suggest that M06 with a triple-zeta (TZ) basis set should be a good choice for studying the energetics of other systems with an overall Si–O–H composition.

**Table 4:** Comparison of DFT Energies to Benchmark CCSD(T) Energies for Reaction Energies for the Formation of Si_2_O_2_, Si_2_O_3_, and Si_2_O_4_ From SiO and SiO_2_ Reactants^a^

		M06	M06	M06-2X	B3LYP	CCSD(T)	CCSD(T)	CCSD(T)
reactant	product	DZ^b^	TZ^c^	TZ^c^	TZ^c^	5Z//M06^d^	5Z//QZ^e^	6Z//QZ^f^
SiO + SiO	Si_2_O_2_	–218.7	–239.2	–256.6	–206.9	–235.5	–235.0	–235.6
SiO + SiO_2_	Si_2_O_3_	–334.1	–354.8	–383.1	–325.9	–356.9		
SiO_2_ + SiO_2_	Si_2_O_4_	–396.5	–420.6	–458.6	–393.6	–431.5		
	MSE	24.9	3.1	–24.8	32.5			
	MUE	24.9	5.6	24.8	32.5			

a Electronic energies relative to the respective reactants are given in kJ/mol.

b 6-31+G(d,p).

c maug-cc-pV(T+d)Z.

d CCSD(T)/aug-cc-pV(5+d)Z//M06/maug-cc-pV(T+d)Z.

e aug-cc-pV(5+d)Z//aug-cc-pV(Q+d)Z.

f aug-cc-pV(6+d)Z//aug-cc-pV(Q+d)Z.

### Reactions of OH with SiO and Si_2_O_2_

**Figure 1:**
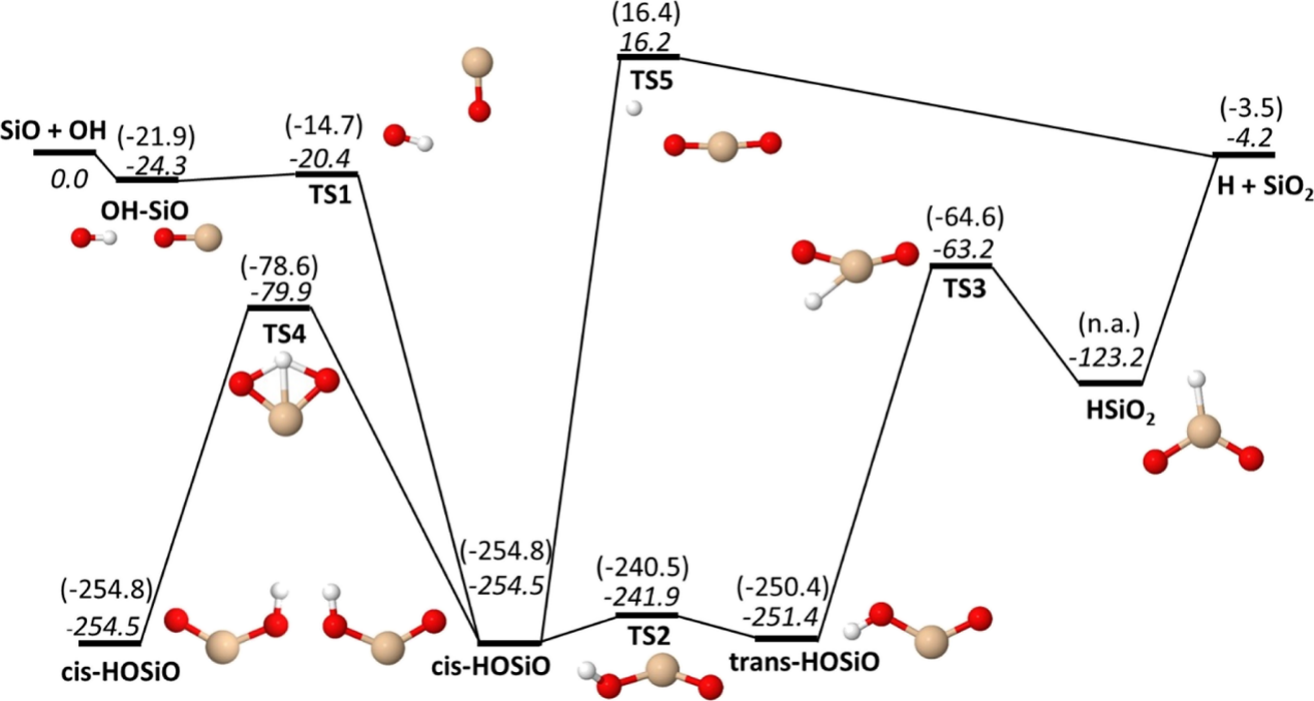
Energetics of the SiO + OH → H + SiO_2_ reaction. Zero-point energy corrected energies relative to SiO + OH are given in kJ/mol for M06/maug-cc-pV(T+d)Z (italic) and CCSD(T)/aug-cc-pV(5+d)Z//M06/maug-cc-pV(T+d)Z (in parentheses).

The highly reactive hydroxyl radical (OH) is found in a wide range of environments, for instance, in combustion systems and in Earth’s atmosphere, where it plays an important role in efficiently removing, for example, various organic molecules, and in the interstellar medium. OH can be formed through a number of different mechanisms, for example, by exposing water (vapor) to high temperatures, UV radiation, and/or electron irradiation. Figure 1 shows a schematic energy diagram of the HOSiO PES including the important reaction pathways of the SiO + OH → H + SiO_2_ reaction. As noted above, the M06/maug-cc-pV(T+d)Z calculations give an excellent description of the energetics of the reaction and it is illustrated here with a direct comparison of relative energies between the M06 results and CCSD(T)/aug-cc-pV(5+d)Z//M06/maug-cc-pV(T+d)Z results. The HSiO_2_ local minimum is an exception, potentially because of strong multireference character in this part of the PES. In a different study, CCSD(T)/aug-cc-pV(Q+d)Z calculations^34^ did locate an optimized HSiO_2_ minimum with *C*_2*v*_ symmetry, whereas M06 finds an asymmetric minimum with *C*_*s*_ symmetry instead. Attempts to calculate the CCSD(T) energy of this *C*_*s*_ geometry failed to converge because a single-reference wavefunction seems inadequate. This problem was only found for this structure and all the other ones were straightforward to calculate using CCSD(T). An analysis using the T1 diagnostic^70−72^ indeed shows that most species on the HOSiO PES should be well described using a single-reference wavefunction, that is, the T1 diagnostic is at most about 0.02 for closed-shell species and well below 0.045 for open-shell species, except for the HSiO_2_ minimum that has a T1 value of 0.089 (see Table S2 in the Supporting Information).

There are no significant barriers on the lowest energy reaction path between reactants and products, but instead the system passes through a significant potential well, meaning that an intermediate HOSiO complex is formed along the course of reaction (*cis*- and *trans*-HOSiO in Figure 1). Using a master-equation approach and a CBS-Q PES, Gomez-Martín et al.^32^ calculated the low-pressure limit of the rate constant for this reaction to be about 5 × 10^–12^ cm^3^ s^–1^ at room temperature. They also found that at room temperature and pressures above 65 Torr, the HOSiO species are sufficiently stabilized to become the major product of the SiO + OH reaction, meaning that at atmospheric pressure, HOSiO formation should also be dominant. It is unclear whether this significantly affects further reaction toward larger clusters or if HOSiO readily reacts with for instance SiO and SiO_2_.

**Figure 2:**
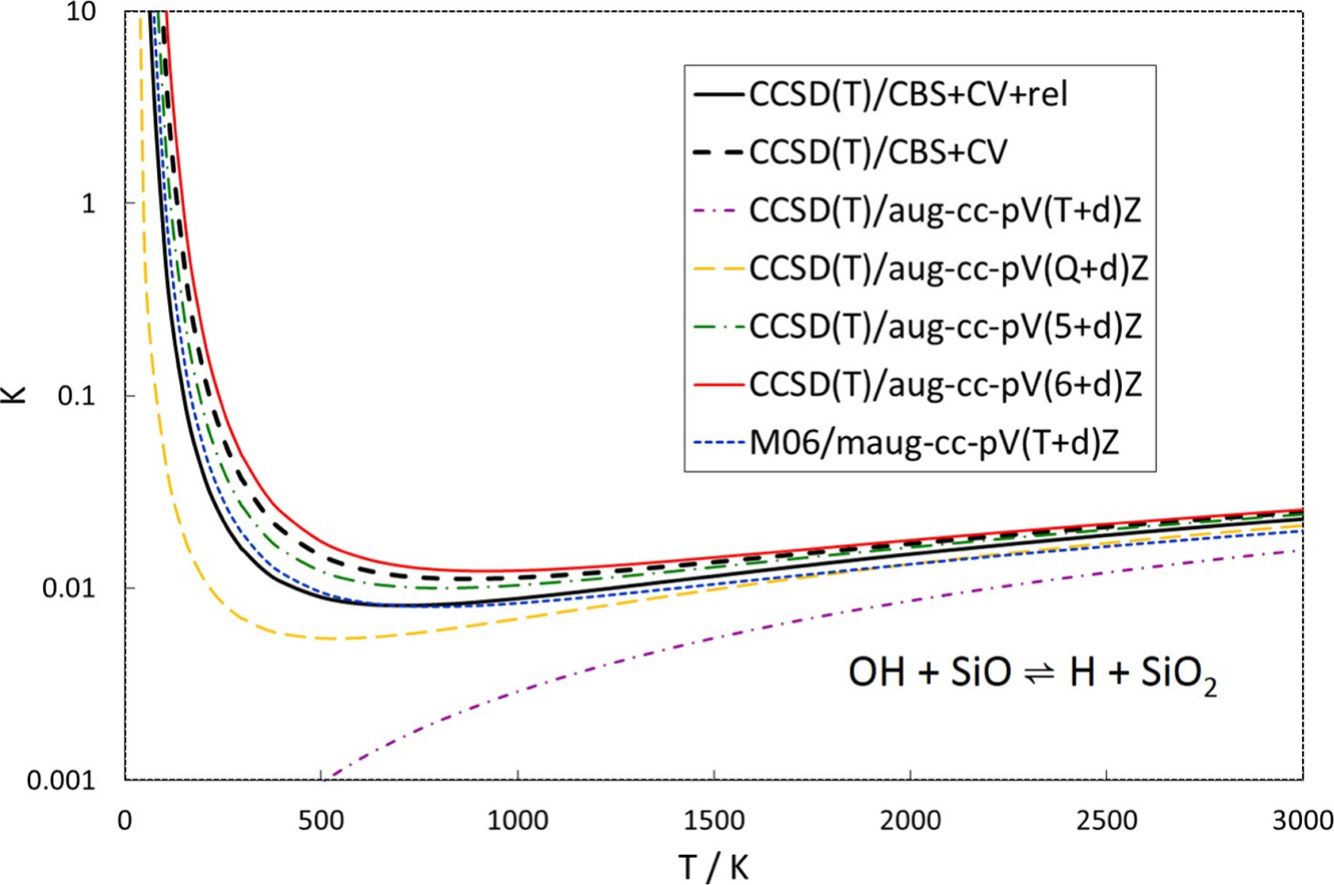
Equilibrium constants of the SiO + OH ⇌ H + SiO_2_ reaction as calculated by CCSD(T) and M06 (see text for details).

An interesting feature of the SiO + OH ⇌ H + SiO_2_ reaction is that it is almost thermoneutral. The electronic energy difference is 7.1 [7.8] kJ/mol as calculated by M06 [CCSD(T)] (see Tables S3 and S4 in the Supporting Information). Adding zero-point and thermal energies, the enthalpy difference is −4.2 [−3.5] kJ/mol at 0 K and −5.0 [-4.2] kJ/mol at room temperature (see Tables S3–S5), meaning that the reaction is slightly exothermic. The free energy difference is 9.8 [10.3] kJ/mol at room temperature (and atmospheric pressure), meaning that the equilibrium constant is shifted toward SiO + OH. Plotting the equilibrium constant over a wide temperature interval (Figure 2) shows that the reaction favors the formation of H + SiO_2_ at very low temperatures, but that OH + SiO should be dominant from around room temperature and above (neglecting any formation of an HOSiO complex). Due to the interesting energetics of the system, the equilibrium constant goes from being ≫1 at very low temperatures, to a minimum on the order of 0.01 and then slowly increasing with the increasing temperature. In Figure 2, we also compare how different levels of theory predict the behavior of the equilibrium. As benchmarks, we have included two sets of complete-basis-set (CBS)-extrapolated CCSD(T) calculations both including core-valence (CV) interactions, but with only one including the relativistic corrections. Since relativistic effects are not included in any of the other calculations, it seems reasonable that they should be compared to benchmark calculations without such effects as well.

The two benchmark calculations differ slightly but not significantly enough to be of much practical importance. What is interesting is that CCSD(T) with a TZ basis set fails to predict the correct behavior of the equilibrium constant at a low temperature, whereas CCSD(T) with QZ, 5Z, and 6Z basis sets and M06/maug-cc-pV(T+d)Z show a very similar behavior to the benchmark values. The reaction free energies underlying the equilibrium constants are given at selected temperatures in Table S5.

**Figure 3:**
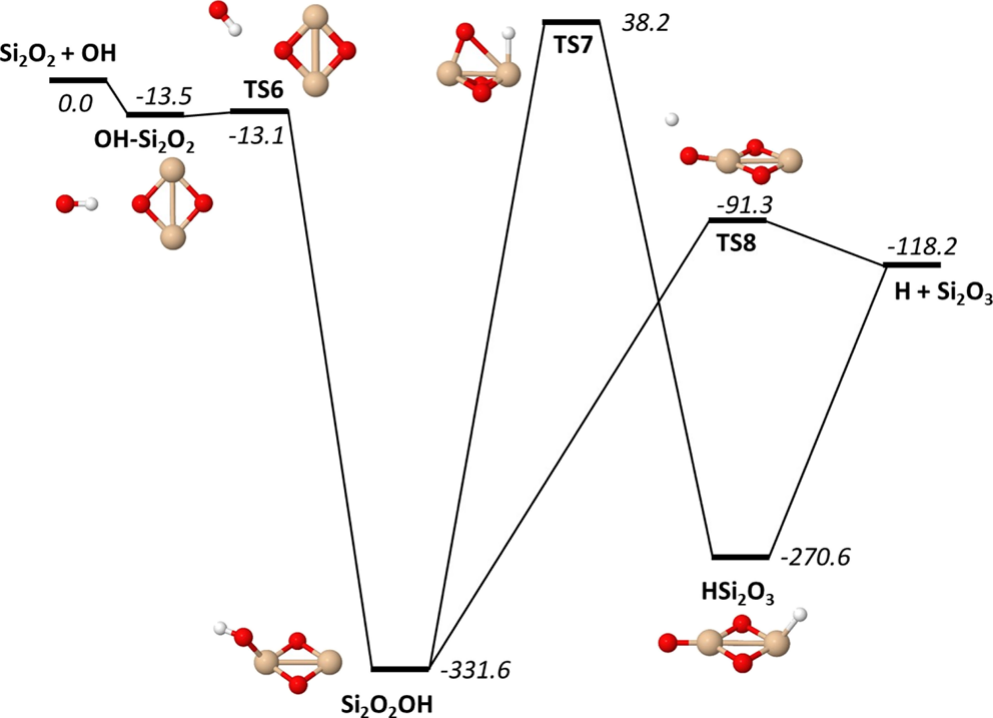
Energetics of the Si_2_O_2_ + OH → H + Si_2_O_3_ reaction. Zero-point energy corrected energies are given in kJ/mol for M06/maug-cc-pV(T+d)Z.

**Figure 4:**
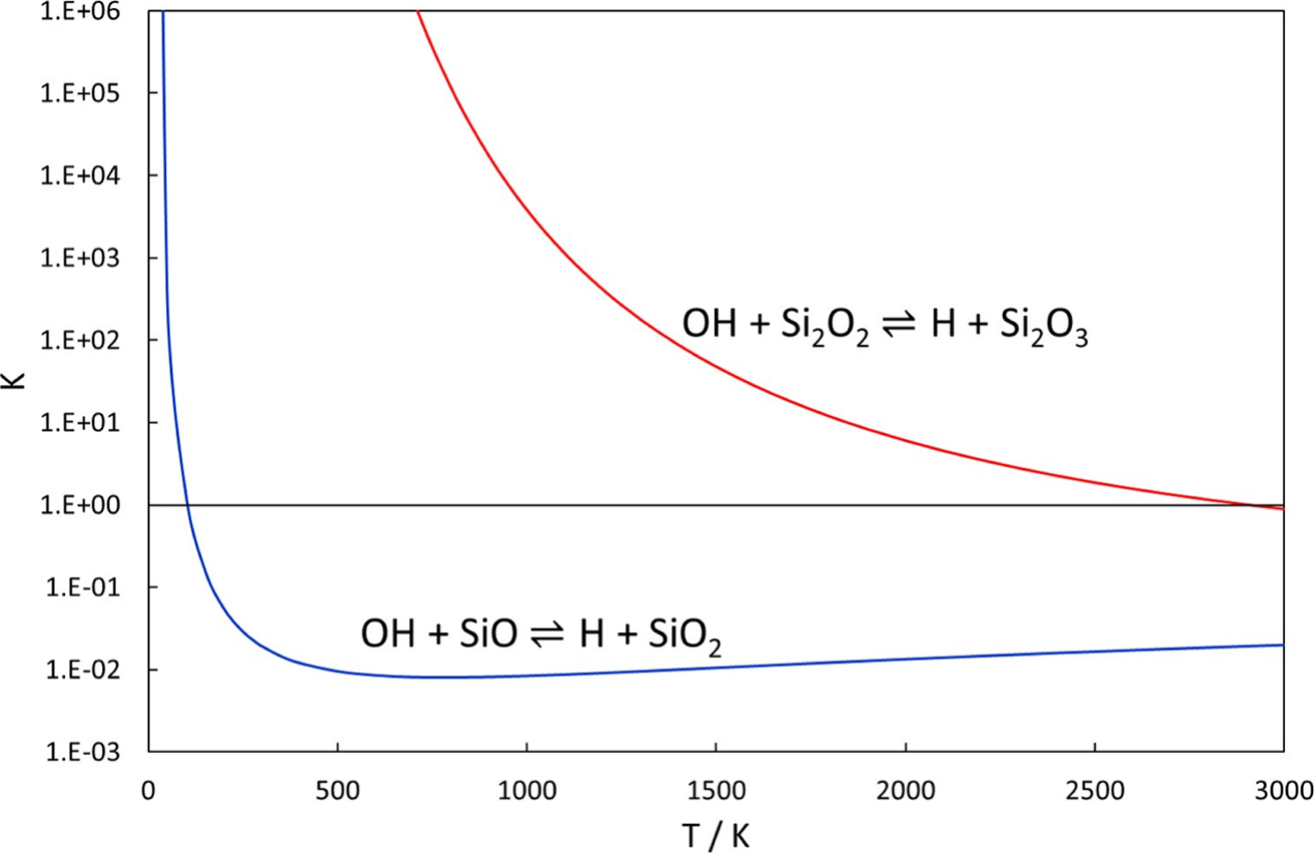
Equilibrium constants of the SiO + OH and Si_2_O_2_ + OH reactions as calculated by M06/maug-cc-pV(T+d)Z.

Considering the reaction of OH with Si_2_O_2_, the PES is similar to HOSiO with some important differences (see Figure 3 and Table S6). The minima are significantly deeper, and more importantly, the reaction to form H and Si_2_O_3_ is exothermic by over 100 kJ/mol. Since there is no significant barrier on the lowest reaction path, one should expect this reaction to be significantly faster than the SiO + OH reaction. A comparison between the equilibrium constants of the two reactions is made in Figure 4. Not surprisingly, the Si_2_O_2_ + OH reaction is strongly shifted toward the H + Si_2_O_3_ products for all temperatures except the very high ones at about 3000 K and above, supporting the abovementioned assumption of a faster reaction than SiO + OH. A detailed analysis of the kinetics of these reactions should be performed to clarify this matter. The reaction free energies underlying the equilibrium constants are given at selected temperatures in Tables S5 and S7.

### Reactions of H_2_O with SiO and Si_2_O_2_

**Figure 5:**
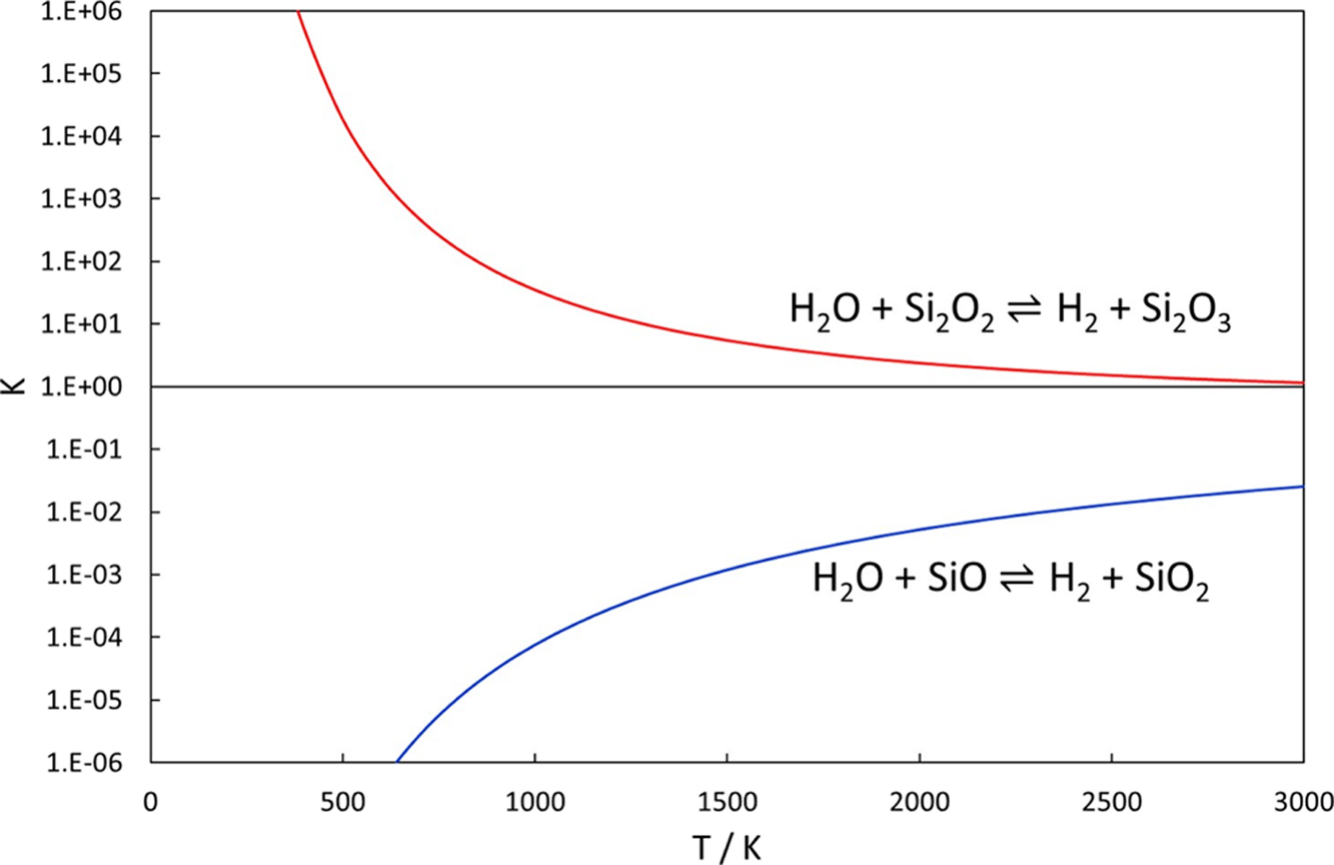
Equilibrium constants of the SiO + H_2_O and Si_2_O_2_ + H_2_O reactions as calculated by M06/maug-cc-pV(T+d)Z.

The reactions of SiO and Si_2_O_2_ with H_2_O might lead to oxidation of the SiO species and formation of H_2_. Inspecting the thermodynamics of the reactions and calculating the equilibrium constants derived from M06 calculations (Figure 5), one finds that the SiO + H_2_O reaction is endothermic by 56.6 kJ/mol and is not driven toward SiO_2_ + H_2_ products at low temperatures. However, at 2000 K, the equilibrium constant is about 0.01 and if the kinetics is fast enough, H_2_O might then oxidize SiO to a significant extent. Upon SiO cluster formation, the reaction thermodynamics becomes completely different. The Si_2_O_2_ + H_2_O → Si_2_O_3_ + H_2_ reaction is *exothermic* by −57.5 kJ/mol, and the chemical equilibrium favors these products at all temperatures. Based on the favorable thermochemistry of this reaction, Goumans and Bromley,^10^ in their analysis of likely mechanisms for the formation of silicate dust in stellar outflows, pointed to this reaction as potentially a key reaction step in going from SiO molecules to Si_2_O_2_ and then on to silicate clusters and particles. However, the kinetics of the Si_2_O_2_ + H_2_O reaction was not analyzed. The reaction free energies underlying the equilibrium constants are given at selected temperatures in Tables S8 and S9.

**Figure 6:**
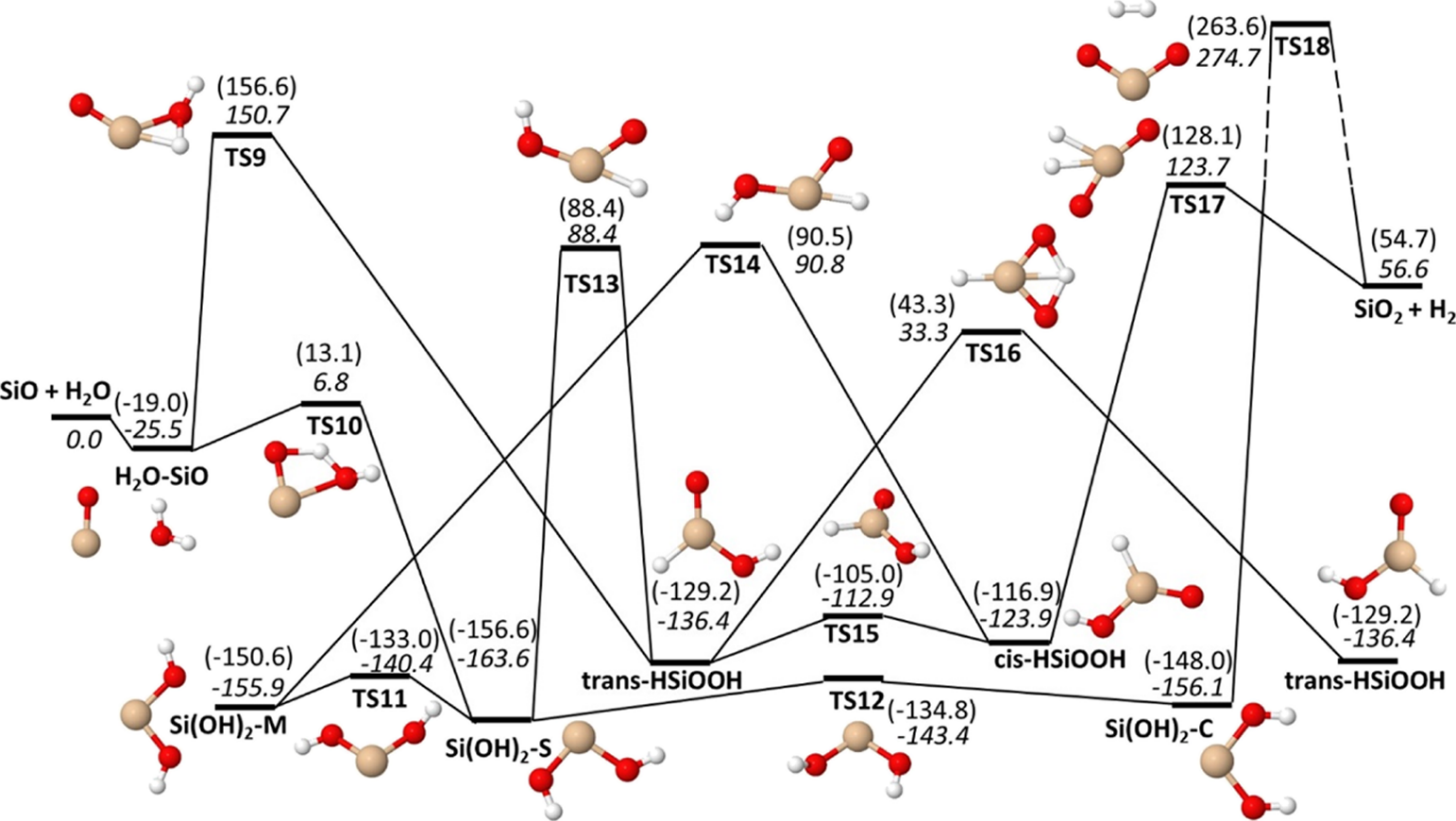
Energetics of the SiO + H_2_O → H_2_ + SiO_2_ reaction. Zero-point energy corrected energies relative to SiO + H_2_O are given in kJ/mol for M06/maug-cc-pV(T+d)Z (italic) and CCSD(T)/aug-cc-pV(5+d)Z//M06/maug-cc-pV(T+d)Z (in parentheses).

Reactions of SiO and Si_2_O_2_ with the stable H_2_O molecule might be expected to be much slower than that with the reactive OH radical. However, inspecting the energetics of the SiO + H_2_O reaction (Figure 6 and Table S10), one finds that there is a barrier (TS10) of only 6.8 [13.1] kJ/mol as calculated by M06 [CCSD(T)] for the formation of a molecular complex that might be relatively long-lived. Initially, this leads to the formation of Si(OH)_2_, which is the most stable species in this reaction system, and it exists in three isomeric forms (S, M, and C) that are connected by low barriers (TS11 and TS12). There is a reaction pathway for dissociating into SiO_2_ + H_2_ directly from Si(OH)_2_, but this has to pass a barrier (TS18) that is 270 kJ/mol above the SiO + H_2_O reactants, making this a much too slow reaction at relevant temperatures. Molecular rearrangement can occur through barriers lying 88–91 kJ/mol over the reactants (TS13 and TS14) to form the HSiOOH formic acid analogue. HSiOOH can also be formed directly from SiO and H_2_O, but the reaction has a barrier of 150 kJ/mol (TS9), making it rather unlikely. If HSiOOH is formed, it might dissociate in SiO_2_ + H_2_ through a barrier (TS17) that is 123.7 kJ/mol above SiO + H_2_O. This barrier is lower than TS9 for HSiOOH formation, so if the reaction occurs directly from SiO + H_2_O, it is not unlikely that it will also dissociate into SiO_2_ + H_2_. However, rearrangement into Si(OH)_2_ has significantly lower barriers and will be more likely. The kinetics of HSiOOH formation through TS9 is then likely an upper limit to the rate of formation of SiO_2_ + H_2_. Stabilization into Si(OH)_2_ and/or redissociation into SiO + H_2_O is much more likely. Analyzing the rates for initially forming HSiOOH vs Si(OH)_2_ will be informative in establishing upper limits to both the overall reaction rate as well as for forming SiO_2_ + H_2_.

**Figure 7:**
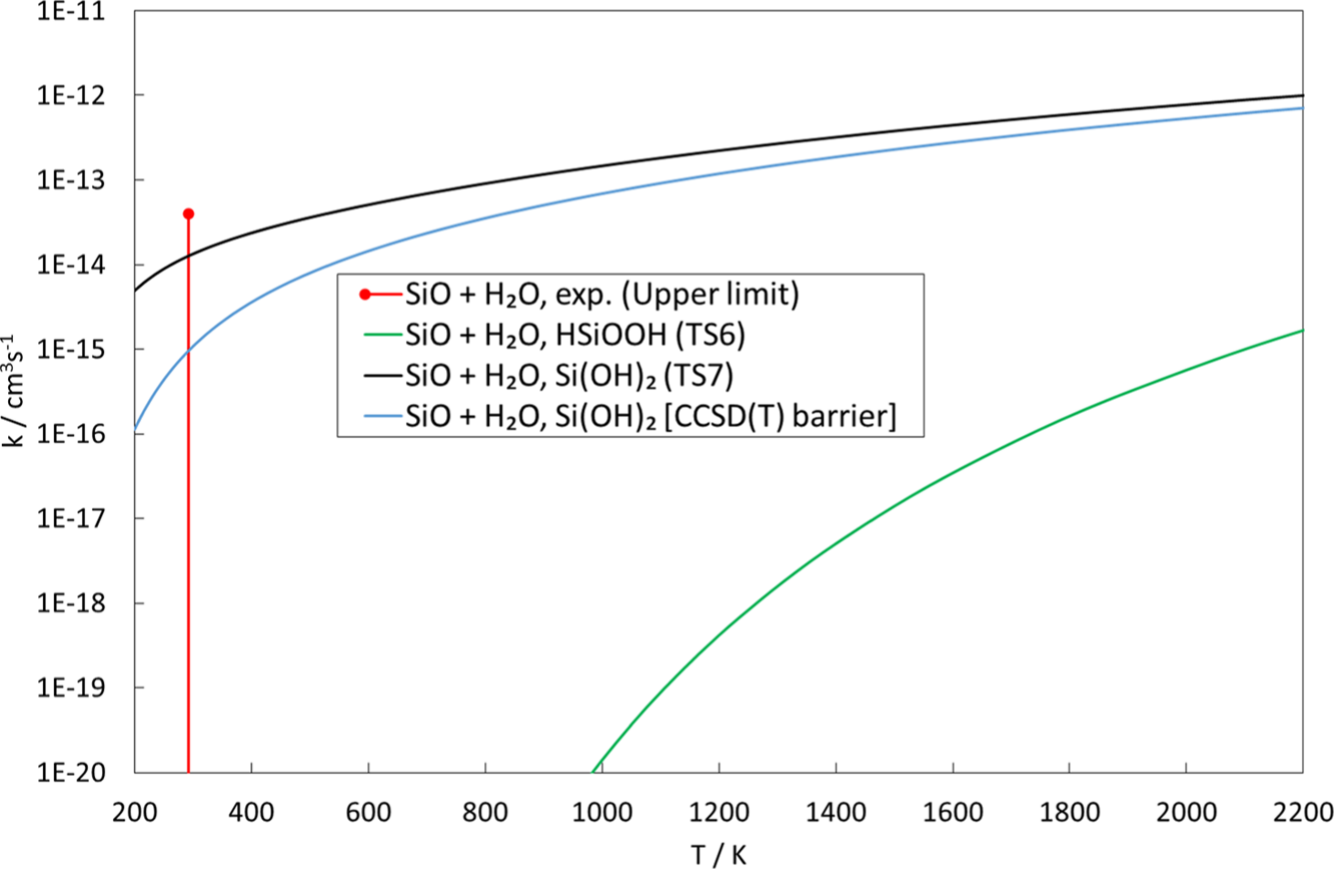
Rate constants of the SiO + H_2_O complex-forming reactions to form HSiOOH and Si(OH)_2_ as calculated by the transition state theory. The experimental upper limit for the overall reaction is taken from ref (32).

Transition state theory calculations of the rate constants for forming HSiOOH and Si(OH)_2_ are presented in Figure 7. The rate calculations are based on M06 calculations but since there is a difference in the barrier height for TS10 of almost 7 kJ/mol between M06 and CCSD(T), both these barrier heights were used to estimate the rate of Si(OH)_2_ formation. In Figure 7, we also compare the rate constants to an experimental upper limit of 4 × 10^–14^ cm^3^ s^–1^ at *T* = 293 K established by Gómez-Martín et al.^32^ Both barriers are consistent with this upper limit. As can be seen, this reaction is moderately fast from low to high temperatures, meaning that the reaction to form Si(OH)_2_ complexes is rather likely, but it is probably too slow to be important at interstellar conditions where temperatures typically are below 100 K (also considering the extremely low pressures, making three-body stabilization of species unlikely). In contrast, formation of HSiOOH is not only thermodynamically but also kinetically unfavorable, having a small rate constant for all temperatures up to 2200 K. This in turn means that the rate of formation of SiO_2_ + H_2_, likely being only a fraction of the rate of forming HSiOOH, should be insignificant at all relevant temperatures and would therefore be kinetically hindered also at 2000 K, preventing chemical equilibrium to be established for this reaction. Regarding the fate of the Si(OH)_2_ species, in the absence of further reaction, it is likely that Si(OH)_2_ is stabilized or redissociates to SiO + H_2_O. In their previous computational work, Zachariah and Tsang^33^ showed that a further addition reaction of Si(OH)_2_ with a H_2_O molecule is possible with a moderate activation energy, forming a HSi(OH)_3_ complex. At the same time, the possible dimerization to form hydrogenated SiO_2_ dimers would have to go through the reaction of two HSiOOH units as also demonstrated by Zachariah and Tsang.^33^ Considering the probably very minor amounts of available HSiOOH, this condensation mechanism seems highly unlikely. One could then conclude that the formation of SiO_2_ dust particles is potentially inhibited if the concentration of gas-phase H_2_O is high relative to SiO. In addition, Plane^11^ found that the reaction of monomeric SiO_2_ with H_2_O leads to the formation of a relatively stable OSi(OH)_2_ complex at a reasonable reaction rate, further hindering the condensation of SiO_2_. If there is an efficient mechanism to dissociate H_2_O to form OH radicals, for example, high temperature and/or presence of reactive radicals, UV photons, or electrons, the situation should become the opposite, accelerating the formation of SiO_2_ particles. Further studies into the mechanisms and kinetics of these processes are necessary to clarify this issue.

**Figure 8:**
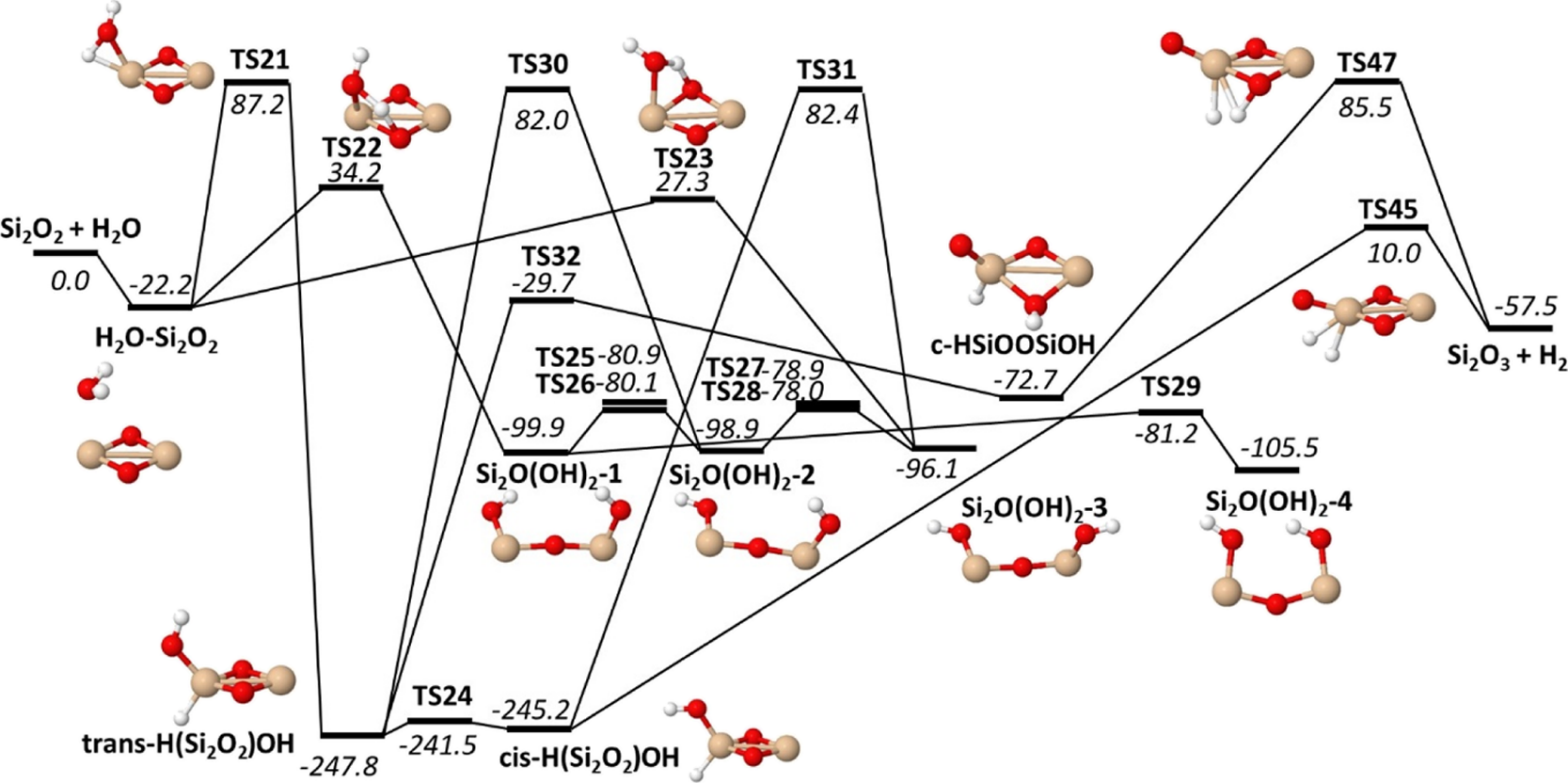
Energetics of the Si_2_O_2_ + H_2_O → H_2_ + Si_2_O_3_ reaction. Only a selected part of the PES is shown. Zero-point energy corrected energies relative to Si_2_O_2_ + H_2_O are given in kJ/mol for M06/maug-cc-pV(T+d)Z.

As discussed above, the reaction of H_2_O with Si_2_O_2_ to form H_2_ with Si_2_O_3_ is exothermic and thermodynamically favorable. The question remains how fast the reaction is. We have identified three different transition states for the initial H_2_O addition to Si_2_O_2_ (Figure 8 and Table S11). There are two with moderate barrier heights around 30 kJ/mol (TS22 and TS23) and one with a high barrier of almost 90 kJ/mol (TS21). Reaction through the lower TS22 and TS23 barriers leads to the formation of a Si_2_O(OH)_2_ species, with four different isomers connected through low barriers (TS25-TS29). Si_2_O(OH)_2_ might rearrange to the more stable H(Si_2_O_2_)OH species, but this requires passing over a relatively high barrier (TS30 or TS31 in Figure 8) that is 82 kJ/mol above the reactant energy. Unless this excess energy is provided to the system from hot reactant molecules, it is unlikely that this rearrangement will occur. The consequence of this is that this molecule might be stabilized as Si_2_O(OH)_2_ until it redissociates into H_2_O and Si_2_O_2_.

The stable *cis*- and *trans*-H(Si_2_O_2_)OH molecules can also be directly formed from H_2_O and Si_2_O_2_ through the higher TS21 barrier. The lowest barrier for forming H_2_ and Si_2_O_3_ products (TS45) is also found for dissociation of *cis*-H(Si_2_O_2_)OH. Since it is only 10 kJ/mol above the reactant energy, this reaction seems quite likely, provided that the H(Si_2_O_2_)OH species are formed. The second lowest transition state for H_2_ + Si_2_O_3_ formation (TS47) is accessed through rearrangement of *trans*-H(Si_2_O_2_)OH into a cyclic c-HSiOOSiOH species through a relative low barrier (TS32) and then dissociation through TS47, which has a high barrier of 85.5 kJ/mol above H_2_O + Si_2_O_2_. There are two additional transition states for H_2_ + Si_2_O_3_ formation that are higher in energy and not shown in Figure 8 since they should be of limited importance. Several other intermediate structures are also omitted from the figure. Information on these can be found in Figure S1 in the Supporting Information.

**Figure 9:**
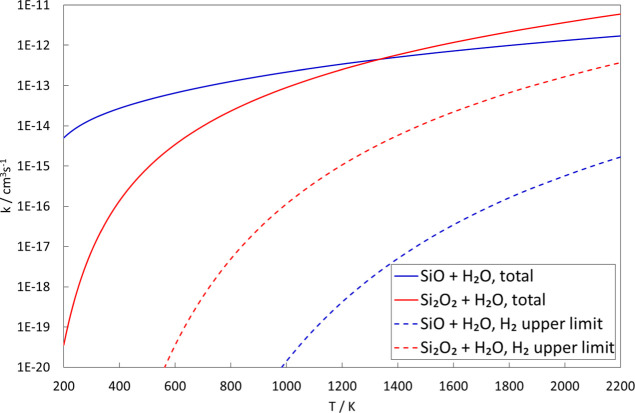
Rate constants of the Si_2_O_2_ + H_2_O complex-forming reactions as calculated by the transition state theory based on M06 calculations compared to those for SiO + H_2_O. The rate of initial formation of *trans*-H(Si_2_O_2_)OH through TS21 is taken as providing the upper limit to the rate of formation of Si_2_O_3_ + H_2_ (see text for details).

Considering the reaction energetics discussed above, it seems reasonable to assume that formation of H_2_ + Si_2_O_3_ has to proceed through the H(Si_2_O_2_)OH minima. It is therefore likely that the direct formation of H(Si_2_O_2_)OH through TS21 and subsequent dissociation through TS45 is the predominant reaction pathway. If one assumes that this provides an upper limit to the rate of formation of H_2_ + Si_2_O_3_, we can compare the calculated rate constants for complex formation in the Si_2_O_2_ + H_2_O and SiO + H_2_O reactions (Figure 9). Due to the higher barriers in the Si_2_O_2_ + H_2_O reaction, complex formation is several orders of magnitude slower at low temperatures than for SiO + H_2_O, meaning that any reaction will be highly unlikely and therefore kinetically hindered, even though production of H_2_ + Si_2_O_3_ should be highly thermodynamically favorable. This would, just as for SiO + H_2_O, preclude any reaction under interstellar conditions. At temperatures above 1000 K, the rates of complex formation for Si_2_O_2_ + H_2_O become comparable to SiO + H_2_O, and at all temperatures, the upper limit to H_2_ formation is higher for Si_2_O_2_ + H_2_O. At high temperatures, this could be a viable reaction pathway even though it seems to be at least 2–3 orders of magnitude less likely than complex formation without further reaction. Whether this is fast enough to be significant might depend on the system at hand, but it is important to note that this reaction will be significantly less efficient than the reaction exothermicity and equilibrium constant would suggest. This might affect the conclusions drawn by Goumans and Bromley^10^ on the importance of the Si_2_O_2_ + H_2_O reaction for dust formation in circumstellar space, even though the temperatures under those conditions often are above 1000 K. As in the case of SiO, the presence of H_2_O might inhibit the formation of larger particles by forming small hydrogenated species that are unlikely to condense to larger clusters. Again, a proper detailed analysis should be made of these reactions and reactions of related species before any definite conclusions can be drawn.

## Conclusions

We reported on computational studies of reactions of SiO and Si_2_O_2_ molecules with OH radicals and H_2_O molecules using electronic structure (DFT and coupled cluster) calculations. The particular interest in these reactions comes from the importance of oxidation of these species for the efficient condensation and formation of larger silica or silicate particles (depending on the environment) in typical temperature ranges between 1000 and 2000 K. For the exploration of reaction mechanisms and reaction energetics, the M06 density functional was identified as having an excellent cost-to-accuracy ratio. For benchmarking the study of Si–O–H reaction systems, we applied CCSD(T) with a pentuple-zeta (5Z) basis set on DFT molecular geometries. We subsequently studied the reactions of SiO and Si_2_O_2_ with OH and H_2_O, respectively. We could conclude that the condensation of SiO into Si_2_O_2_ significantly affects reactivity and it was suggested, based on DFT results, that the highly exothermic reaction of Si_2_O_2_ with OH radicals to form H + Si_2_O_3_ should be significantly faster than the almost thermoneutral SiO + OH → H + SiO_2_ reaction. This latter reaction has already previously been found, both experimentally and theoretically, to be rather efficient. The corresponding reactions with H_2_O showed a similar trend. The endothermic SiO + H_2_O → H_2_ + SiO_2_ was not expected to be an efficient reaction for SiO oxidation, but it was demonstrated that the high energy barriers of the system basically made the kinetics even less efficient than expected from thermodynamic equilibrium considerations. Instead intermediate Si(OH)_2_ complexes were argued to be the most probable products. The exothermic Si_2_O_2_ + H_2_O → H_2_ + Si_2_O_3_ reaction has been expected to be efficient based on the reaction thermodynamics. However, there are significant barriers to reaction also in this system, which seemed to suggest that the most likely products are meta-stable Si_2_O(OH)_2_ complex species, even though the formation of H_2_ and Si_2_O_3_ does seem much more likely than the corresponding reaction between SiO and H_2_O. This could then still be a viable oxidation reaction, at least at higher temperatures, albeit less efficient than one might expect. Based on these results, it is questionable whether H_2_O vapor might lead to significant oxidation of SiO and SiO clusters. If the conditions are such that H_2_O dissociates to form OH radicals, then SiO oxidation and subsequent particle formation might be accelerated instead.

To be able to make more definite statements on the kinetics of these reactions, we perform detailed analyses of the kinetics of the reactions using RRKM calculations that will be presented in a separate publication.

## Supporting Information Available

Additional stationary points on the Si_2_O_2_ + H_2_O PES, calculated and experimental geometric and spectroscopic data of small molecules, energy (electronic and zero-point energy corrected), enthalpy, and free energy differences of reactants and products, stationary points on the PESs, and coordinates of all stationary points (PDF)
