# Supplementary material for: Mechanisms and Thermochemistry of Reactions of SiO and Si2O2 with OH and H2O
Source: J Phys Chem A. 2023 May 2;127(18):4015–26. doi: 10.1021/acs.jpca.3c00862 (PMC10184121; doi:10.1021/acs.jpca.3c00862)
Supplement: Supplementary file 1 — jp3c00862_si_001.pdf [file jp3c00862_si_001.pdf]

Supporting information

Mechanisms and Thermochemistry of Reactions of SiO and Si_2O_2 with OH and H_2O

Stefan Andersson

Department of Metal Production and Processing, SINTEF, P.O. Box 4760 Torgarden, 7465

Trondheim, Norway

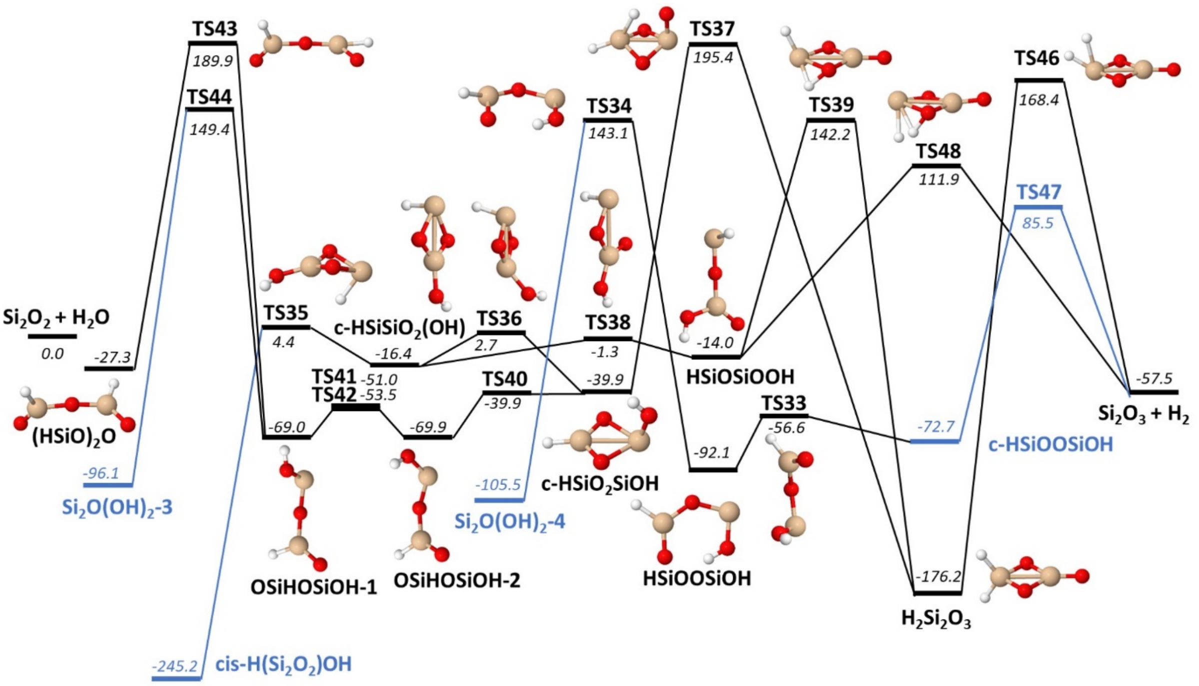

Figure S1. High-energy part of the potential energy surface for the $Si_2O_2 + H_2O \rightarrow H_2 + Si_2O_3$ reaction. Zero-point energy corrected energies relative to $Si_2O_2 + H_2O$ are given in kJ/mol for M06/maug-cc-pV(T+d)Z. Stationary points already included in Figure 8 of the main paper are marked by blue color.

Table S1

Structural parameters and vibrational frequencies of stable molecules. Bond distances are in Å, angles in degrees and frequencies in cm^{-1}.

Molecule	Parameter	CCSD(T) aug-cc-pV(Q+d)Z	M06 maug-cc-pV(T+d)Z	Experimental
SiO	R_{SiO}	1.518	1.520	1.510[a]
	ω	1233	1262	1242[b]
SiO_2	R_{SiO}	1.514	1.511	-
	ω_1	289	307	273[c]
	ω_2	289	307	273[c]
	ω_3	987	1039	-
	ω_4	1432	1504	1416[c]
Si_2O_2	R_{SiO}	1.695	1.686	1.71[d]
	α(O-Si-O)	87.5	87.3	87[d]
	ω_1	237	242	-
	ω_2	558	560	-
	ω_3	578	584	-
	ω_4	780	796	766[d]
	ω_5	824	839	805[d]
	ω_6	856	876	-
OH	R_{OH}	0.971	0.970	0.970[e]
	ω	3739	3771	3738[f]
H_2O	R_{OH}	0.959	0.957	0.958[g]
	α	104.4	105.1	104.5[g]
	ω_1	1650	1611	1649[h]
	ω_2	3831	3890	3832[h]
	ω_3	3940	4012	3943[h]

[a]Sanz et al. (2003), [b]Beer et al. (1974), [c]Andrews and McCluskey (1992), [d]Anderson and Ogden (1969), [e]Amano (1984), [f]Maillard et al. (1976), [g]Jensen et al. (1994), [h]Benedict et al. (1956)

References

Amano, T. Difference Frequency Laser Spectroscopy of OH and OD: Simultaneous Fit of the Infrared and Microwave Lines. *J. Mol. Spectrosc.* **1984**, *103*, 436 – 454.

Anderson, J. S.; Ogden, J. S. Matrix Isolation Studies of Group-IV Oxides. I. Infrared Spectra and Structures of SiO, Si_2O_2, and Si_3O_3. *J. Chem. Phys.* **1969**, *51*, 4189 – 4196.

Andrews, L.; McCluskey, M. Bending Modes of SiO_2 and GeO_2 in Solid Argon. *J. Mol. Spectrosc.* **1992**, *154*, 223 -225.

Beer, R.; Lambert, D. L.; Sneden, C. The Silicon Monoxide Radical and the Atmosphere of α Orionis. *Pub. Astron. Soc. Pacific*, **1974**, *86*, 806 – 812.

Benedict, W. S.; Gailar, N.; Plyler, E. K. Rotation-Vibration Spectra of Deuterated Water Vapor. *J. Chem. Phys.* **1956**, *24*, 1139 – 1165.

Jensen, P.; Tashkun, S. A.; Tyuterev, V. G. Refined Potential Energy Surface for the Electronic Ground State of the Water Molecule. *J. Mol. Spectrosc.* **1994**, *168*, 271 – 289.

Maillard, J. P.; Chauville, J.; Mantz, A. W. High-Resolution Emission Spectrum of OH in an Oxyacetylene Flame from 3.7 to 0.9 μm. *J. Mol. Spectrosc.* **1976**, *63*, 120 – 141.

Sanz, M. E.; McCarthy, M. C.; Thaddeus, P. Rotational transitions of SO, SiO, and SiS excited by a discharge in a supersonic molecular beam: Vibrational temperatures, Dunham coefficients, Born–Oppenheimer breakdown, and hyperfine structure. *J. Chem. Phys.* **2003**, *119*, 11715 – 11727.

Table S2. T1 diagnostic of stationary points on the HOSiO potential energy surface based on CCSD/aug-cc-pV(5+d)Z calculations on geometries optimized using M06/maug-cc-pV(T+d)Z.

**Species**	**T1 diagnostic**
SiO	0.024
OH	0.008
SiO_2	0.022
OH-SiO	0.019
TS1	0.019
cis-HOSiO	0.020
TS2	0.020
trans-HOSiO	0.021
TS3	0.029
$HSiO_2$	0.089*
TS4	0.022
TS5	0.022

*calculation did not converge

Table S3. Electronic energies for CCSD(T) and M06, and enthalpy and Gibbs free energy differences for stationary points (minima and transition states) on the potential energy surface for the SiO + OH reaction. Energies relative to SiO + OH are given in kJ/mol.

	CCSD(T)/5Z//M06	M06/TZ	M06/TZ	M06/TZ	M06/TZ
	ΔE	ΔE	ΔH(0 K)	ΔH(298 K)	ΔG(298 K)
SiO + OH	0.0	0.0	0.0	0.0	0.0
H + SiO_2	7.8	7.1	-4.2	-5.0	9.8
OH-SiO	-29.0	-31.4	-24.3	-26.5	3.3
TS1	-20.2	-25.9	-20.4	-25.4	7.8
cis-HOSiO	-267.8	-268.5	-254.4	-260.0	-223.0
TS2	-251.7	-253.1	-241.9	-248.2	-210.1
trans-HOSiO	-265.1	-265.8	-251.4	-257.0	-220.1
TS3	-62.9	-61.5	-63.2	-69.1	-30.9
$HSiO_2$	n.a.[f]	-126.9	-123.2	-128.2	-92.7
TS4	-83.6	-84.9	-79.9	-87.2	-46.0
TS5	27.6	27.4	16.2	12.3	47.3

Table S4. Electronic energy, enthalpy and free energy differences (in kJ/mol) between SiO + OH and other gas-phase species on the HOSiO potential energy surface as calculated by M06/maug-cc-pV(T+d)Z. Free energies are calculated for a pressure of 1 atm.

	Δ*E*	Δ*H*(0 K)	Δ*H*(298 K)	Δ*G*(298 K)	Δ*G*(1000 K)	Δ*G*(2000 K)
SiO + OH	0.0	0.0	0.0	0.0	0.0	0.0
H + SiO_2	7.1	-4.2	-5.0	9.8	39.8	71.8
OH-SiO	-31.4	-24.3	-26.5	2.1	65.5	146.3
TS1	-25.9	-20.4	-25.4	7.8	87.5	201.8
cis-HOSiO	-268.5	-254.5	-260.1	-223.2	-136.0	-16.4
TS2	-253.1	-241.9	-248.2	-210.1	-116.9	18.0
trans-HOSiO	-265.8	-251.4	-257.1	-219.9	-132.3	-11.9
TS3	-61.5	-63.2	-69.1	-30.9	61.5	192.6
$HSiO_2$	-126.9	-123.2	-128.2	-92.7	-8.6	104.6
TS4	-84.9	-79.9	-87.2	-46.0	56.4	203.9
TS5	27.4	16.2	12.3	47.3	128.9	241.6

Table S5. Reaction enthalpy and free energy (in kJ/mol) for SiO + OH → H + SiO_2 as calculated by M06/maug-cc-pV(T+d)Z and CCSD(T)/CBS with and without relativistic corrections. Free energies are calculated for a pressure of 1 atm.

T / K	Δ*H* M06	Δ*H* CCSD(T) non-rel	Δ*H* CCSD(T) relativistic	Δ*G* M06	Δ*G* CCSD(T) non-rel	Δ*G* CCSD(T) relativistic
0	-4.2	-5.6	-3.5	-4.2	-5.6	-3.5
100	-5.1	-6.5	-4.5	-0.2	-1.7	0.4
298.15	-5.0	-6.3	-4.2	9.8	8.2	10.3
1000	2.8	1.8	3.9	39.8	37.3	39.3
2000	15.2	14.2	16.3	71.8	67.7	69.8
3000	25.7	24.8	26.9	97.8	92.2	94.2

Table S6. Electronic energy and zero-point energy corrected energy differences (in kJ/mol) between Si_2O_2 + OH and other gas-phase species on the ground state potential energy surface as calculated by M06/maug-cc-pV(T+d)Z. Free energies are calculated for a pressure of 1 atm.

	Δ*E*	Δ*H*(0 K)
OH + Si_2O_2	0.0	0.0
H + Si_2O_3	-108.5	-118.2
OH-Si_2O_2	-20.0	-13.5
TS6	-18.2	-13.1
Si_2O_2OH	-348.0	-331.6
TS7	35.4	38.2
HSi_2O_3	-282.8	-270.6
TS8	-83.1	-91.3

Table S7. Reaction enthalpy and free energy (in kJ/mol) for Si_2O_2 + OH → H + Si_2O_3 as calculated by M06/maug-cc-pV(T+d)Z. Free energies are calculated for a pressure of 1 atm.

T / K	Δ*H*	Δ*G*
	M06	M06
0	-118.2	-118.2
100	-119.1	-113.8
298.15	-119.2	-102.9
1000	-112.2	-68.7
2000	-100.1	-29.9
3000	-89.7	2.8

Table S8. Reaction enthalpy and free energy (in kJ/mol) for $SiO + H_2O \rightarrow H_2 + SiO_2$ as calculated by M06/maug-cc-pV(T+d)Z and CCSD(T)/CBS with and without relativistic corrections. Free energies are calculated for a pressure of 1 atm.

T / K	Δ*H* M06	Δ*H* CCSD(T) non-rel	Δ*H* CCSD(T) relativistic	Δ*G* M06	Δ*G* CCSD(T) non-rel	Δ*G* CCSD(T) relativistic
0	56.6	54.2	55.7	56.6	54.2	55.7
100	56.3	53.9	55.4	59.6	57.3	58.7
298.15	57.5	55.3	56.8	65.8	63.3	64.8
1000	66.4	64.5	65.9	78.8	75.4	76.9
2000	76.0	74.0	75.5	87.1	82.3	83.7
3000	82.8	80.7	82.1	91.2	84.9	86.3

Table S9. Reaction enthalpy and free energy (in kJ/mol) for $Si_2O_2 + H_2O \rightarrow H_2 + Si_2O_3$ as calculated by M06/maug-cc-pV(T+d)Z. Free energies are calculated for a pressure of 1 atm.

T / K	Δ*H* M06	Δ*G* M06
0	-57.5	-57.5
100	-57.7	-53.9
298.15	-57.0	-46.9
1000	-48.6	-29.7
2000	-39.3	-14.6
3000	-32.6	-3.8

Table S10. Electronic energy, enthalpy and free energy differences (in kJ/mol) between SiO + H_2O and other gas-phase species as calculated by M06/maug-cc-pV(T+d)Z. Free energies are calculated for a pressure of 1 atm.

	ΔE	ΔH(0 K)	ΔH(298 K)	ΔG(298 K)	ΔG(1000 K)	ΔG(2000 K)
SiO + H_2O	0.0	0.0	0.0	0.0	0.0	0.0
H_2 + SiO_2	76.4	56.6	57.5	65.7	78.8	87.1
H_2O-SiO	-31.9	-25.5	-27.5	1.7	65.7	146.7
TS9	159.7	150.7	145.6	183.1	269.7	386.1
TS10	8.0	6.8	0.3	39.9	134.0	263.4
$Si(OH)_2$-S	-173.3	-163.6	-169.3	-130.7	-43.3	70.5
TS11	-146.1	-140.4	-146.3	-107.3	-16.5	108.7
$Si(OH)_2$-M	-165.9	-155.9	-161.5	-121.2	-30.1	89.3
TS12	-149.0	-143.4	-149.7	-110.8	-20.3	104.3
$Si(OH)_2$-C	-163.9	-156.1	-161.2	-121.9	-33.5	81.2
TS13	95.9	88.4	83.9	120.0	203.2	315.1
TS14	98.0	90.8	86.2	122.8	207.0	320.3
trans-HSiOOH	-141.5	-136.4	-142.6	-102.9	-11.1	109.1
TS15	-113.9	-112.9	-119.4	-79.0	16.7	149.0
cis-HSiOOH	-128.1	-123.9	-129.8	-90.4	0.2	118.6
TS16	38.5	33.3	25.9	69.3	174.2	319.0
TS17	132.9	123.7	117.2	156.7	251.4	380.7
TS18	292.1	274.7	268.7	309.7	405.3	532.3

Table S11. Electronic energy and zero-point energy corrected energy differences (in kJ/mol) between Si_2O_2 + H_2O and other gas-phase species on the ground state potential energy surface as calculated by M06/maug-cc-pV(T+d)Z. Free energies are calculated for a pressure of 1 atm.

	Δ*E*	Δ*H*(0 K)
H_2O + Si_2O_2	0.0	0.0
H_2 + Si_2O_3	-39.2	-57.5
H_2O-Si_2O_2	-29.1	-22.2
TS21	91.5	87.2
TS22	34.6	34.2
TS23	27.0	27.3
trans-H(Si_2O_2)OH	-255.8	-247.8
TS24	-247.5	-241.5
cis-H(Si_2O_2)OH	-251.8	-245.2
$Si_2O(OH)_2$-1	-106.4	-99.9
TS25	-83.2	-80.9
TS26	-82.5	-80.1
$Si_2O(OH)_2$-2	-106.0	-98.9
TS27	-81.6	-78.9
TS28	-80.8	-78.0
$Si_2O(OH)_2$-3	-103.7	-96.1
TS29	-85.3	-81.2
$Si_2O(OH)_2$-4	-114.6	-105.5
TS30	86.4	82.0
TS31	86.0	82.4
TS32	-26.0	-29.7
c-HSiOOSiOH	-77.6	-72.7
TS33	-54.9	-56.6
HSiOOSiOH	-92.1	-87.4
TS34	151.1	143.1
TS35	4.0	4.4
c-HSiSiO_2(OH)	-18.9	-16.4
TS36	2.7	2.7
c-HSiO_2SiOH	-39.8	-39.9
TS37	204.3	195.4
$H_2Si_2O_3$	-180.7	-176.2
TS38	-2.3	-1.3
HSiOSiOOH	-14.3	-14.0
TS39	150.9	142.2
TS40	-39.7	-39.9
OSiHOSiOH-1	-71.3	-69.0
TS41	-48.5	-51.0
TS42	-50.8	-53.5
OSiHOSiOH-2	-71.8	-69.9
TS43	203.5	189.9
$(HSiO)_2O$	-24.3	-27.3
TS44	158.2	149.4
TS45	15.3	10.0
TS46	178.4	168.4
TS47	90.5	85.5
TS48	119.4	111.9

Molecular structures (optimized at M06/maug-cc-pV(T+d)Z level)

SiO + OH

OH-SiO

4

OH-OSi M06/maug-cc-pV(T+d)Z

H	-0.000000	0.000000	-1.745566
O	0.000000	-0.000000	-2.727213
Si	0.000000	-0.000000	1.623068
O	-0.000000	0.000000	0.117919

cis-HOSiO

4

cis-HOSiO M06/maug-cc-pV(T+d)Z

H	-1.184268	-1.494461	-0.000007
O	-1.306028	-0.539917	0.000012
Si	0.001523	0.415687	-0.000010
O	1.455764	-0.015860	0.000005

trans-HOSiO

4

trans-HOSiO M06/maug-cc-pV(T+d)Z

H	-2.120195	-0.383136	0.000028
O	-1.208487	-0.680030	-0.000027
Si	0.003415	0.400458	-0.000020
O	1.473992	0.045057	0.000019

$HSiO_2$

4

HSiO2 M06/maug-cc-pV(T+d)Z

H	-0.178175	1.818425	0.000003
Si	0.024923	0.369993	-0.000009
O	1.342656	-0.391172	0.000003

O -1.323912 -0.477195 0.000003

TS1

4

TS1 M06/maug-cc-pV(T+d)Z

H -0.831965 -1.372519 0.011581

O -1.777832 -1.126928 0.021269

Si 0.743001 1.184874 -0.021512

O 0.833788 -0.319977 -0.011339

TS2

4

TS2 M06/maug-cc-pV(T+d)Z

H -1.965002 -0.471054 0.620980

O -1.378229 -0.238759 -0.098839

Si 0.113461 0.376443 0.014653

O 1.437442 -0.357747 -0.002278

TS3

4

TS3 M06/maug-cc-pV(T+d)Z

H -1.490421 0.737578 -0.000000

O -1.307865 -0.699939 -0.000000

Si 0.001181 0.179338 0.000001

O 1.495755 0.297624 -0.000001

TS4

4

TS4 M06/maug-cc-pV(T+d)Z

H 0.000000 0.000000 -1.048789

Si 0.000000 0.000000 0.583417

O -0.000000 1.188714 -0.443619

O -0.000000 -1.188714 -0.443619

TS5

4

TS5 M06/maug-cc-pV(T+d)Z

H -0.933269 -2.796732 0.000000

O -0.958059 -1.044514 -0.000000

Si -0.001485 0.126936 0.000001

O 1.073910 1.169624 -0.000000

Si_2O_2 + OH

OH-Si_2O_2

6

Si2O2-OH M06/maug-cc-pV(T+d)Z

O 0.007421 1.251234 0.008651

Si 0.000469 0.072603 1.231927

O -0.006262 -1.067227 -0.004792

Si 0.000740 0.086955 -1.228118

H 0.038857 3.162690 0.035517

O 0.058774 4.141774 0.056814

Si_2O_2OH

6

trans-Si2O2OH M06/maug-cc-pV(T+d)Z

Si 0.544845 -0.307765 -0.792213

O 2.044836 -0.061907 -0.215107

O -0.478719 -1.084778 0.282103

O -0.358941 1.079492 -0.585304

Si -1.387554 0.313634 0.505573

H 2.773186 -0.624265 -0.473307

HSi_2O_3

6

HSi2O3 M06/maug-cc-pV(T+d)Z

Si	-0.276363	0.739366	0.341182
O	-0.330617	0.942097	1.821965
O	-1.399060	0.504851	-0.834300
O	0.928849	0.683244	-0.772861
Si	-0.193202	0.464126	-2.012511
H	-0.072104	-0.891238	-2.616799

TS6

6

TS6 M06/maug-cc-pV(T+d)Z

O	-0.002271	1.256531	-0.367616
Si	0.005731	0.424683	1.114617
O	0.002104	-0.990264	0.203430
Si	-0.005410	-0.178732	-1.270343
H	0.035963	3.169216	-0.073029
O	0.063882	3.966595	0.492941

TS7

6

TS7 M06/maug-cc-pV(T+d)Z

Si	0.870064	-0.288360	-0.121374
O	0.918000	1.325325	-0.385868
O	-0.214106	-0.198717	1.271972
O	-0.615543	-0.565261	-1.039030
Si	-1.152241	0.462220	0.110594
H	-0.607662	1.877593	-0.207347

TS8

6

TS8 M06/maug-cc-pV(T+d)Z

Si	0.692283	0.084157	-0.207509
O	2.184423	0.311852	-0.111687
O	-0.344427	-0.996952	0.462204
O	-0.574201	1.002932	-0.701462
Si	-1.692697	-0.085573	-0.028407
H	2.872273	1.123305	1.147735

$SiO + H_2O$

H_2O-SiO

5

H2O-SiO M06/maug-cc-pV(T+d)Z

H	1.391754	0.872782	-0.008878
O	1.724514	-0.028600	-0.113197
H	2.522265	-0.108114	0.413240
Si	-0.733629	-0.565389	0.013315
O	-0.858393	0.942975	0.024825

$Si(OH)_2$-M

5

Si(OH)2-M M06/maug-cc-pV(T+d)Z

H	0.000078	2.121431	-0.147060
O	-0.000026	1.232198	-0.501538
Si	-0.000059	0.000000	0.588429
O	-0.000137	-1.232198	-0.501538
H	0.000142	-2.121431	-0.147060

Si(OH)$_2$-S

5

Si(OH)2-M M06/maug-cc-pV(T+d)Z

H	0.000078	2.121431	-0.147060
O	-0.000026	1.232198	-0.501538
Si	-0.000059	0.000000	0.588429
O	-0.000137	-1.232198	-0.501538
H	0.000142	-2.121431	-0.147060

Si(OH)$_2$-C

5

Si(OH)2-C M06/maug-cc-pV(T+d)Z

H	-0.000014	1.252927	-1.340818
O	0.000013	1.307612	-0.383375
Si	-0.000000	0.000000	0.619035
O	-0.000013	-1.307612	-0.383375
H	0.000014	-1.252927	-1.340818

trans-HSiOOH

5

trans-HSiOOH M06/maug-cc-pV(T+d)Z

H	-1.081411	-1.591715	0.000032
O	-1.253965	-0.647686	-0.000039
Si	0.003319	0.358167	-0.000182
O	1.464208	0.004355	0.000063
H	-0.588800	1.694762	0.000125

cis-HSiOOH

5

cis-HSiOOH M06/maug-cc-pV(T+d)Z

H	-2.067658	-0.539571	0.000009
O	-1.145259	-0.798371	0.000000

Si	-0.000318	0.332485	-0.000042
O	1.478120	0.076343	0.000020
H	-0.641482	1.653746	0.000013

TS9

5

TS9 M06/maug-cc-pV(T+d)Z

H	1.848086	1.052942	0.312862
O	1.421989	0.271376	-0.080928
Si	-0.206045	-0.417602	-0.010064
O	-1.434417	0.456597	0.016925
H	1.323810	-0.974470	0.292611

TS10

5

TS10/maug-cc-pV(T+d)Z

H	2.022052	-0.038802	0.553476
O	1.342241	0.053991	-0.119113
Si	-0.494467	-0.612912	0.012492
O	-0.757573	0.929306	0.027763
H	0.610327	0.910227	-0.006343

TS11

5

TS11 M06/maug-cc-pV(T+d)Z

H	1.698113	0.002753	-1.380055
O	1.307682	0.004509	-0.504473
Si	-0.337969	0.109038	-0.433810
O	-0.571746	-0.055783	1.173979
H	-0.712679	0.571303	1.877839

TS12

5

TS12 M06/maug-cc-pV(T+d)Z

H	0.177353	0.204647	1.748556
O	0.850885	0.099615	1.071416
Si	0.397787	-0.077355	-0.498729
O	-1.247488	-0.000857	-0.430700
H	-1.861879	0.727832	-0.471322

TS13

5

TS13 M06/maug-cc-pV(T+d)Z

H	-1.632163	1.101488	-0.004949
O	-1.453114	0.156210	0.021068
Si	0.068450	-0.380089	0.041229
O	1.343406	0.506905	0.019146
H	1.456074	-1.020589	0.079670

TS14

5

TS14 M06/maug-cc-pV(T+d)Z

H	-2.040241	-0.946133	0.027621
O	-1.436181	-0.202117	0.025115
Si	0.167911	-0.397878	0.012759
O	1.145815	0.803400	0.007587
H	1.679996	-0.637122	0.031789

TS15

5

TS15 M06/maug-cc-pV(T+d)Z

H	-2.013266	-0.470075	0.583321
O	-1.356040	-0.302458	-0.086102

Si	0.115429	0.323080	0.010425
O	1.419807	-0.421451	0.004943
H	0.003799	1.783852	0.014725

TS16

5

TS16 M06/maug-cc-pV(T+d)Z

H	-1.632163	1.101488	-0.004949
O	-1.453114	0.156210	0.021068
Si	0.068450	-0.380089	0.041229
O	1.343406	0.506905	0.019146
H	1.456074	-1.020589	0.079670

TS17

5

TS17 M06/maug-cc-pV(T+d)Z

H	2.022052	-0.038802	0.553476
O	1.342241	0.053991	-0.119113
Si	-0.494467	-0.612912	0.012492
O	-0.757573	0.929306	0.027763
H	0.610327	0.910227	-0.006343

TS18

5

TS18 M06/maug-cc-pV(T+d)Z

H	-0.451390	-1.612720	-0.000000
O	-1.304723	-0.291421	-0.000000
Si	-0.000007	0.548152	0.000001
O	1.304436	-0.291849	-0.000001
H	0.450690	-1.612854	0.000001

Si_2O_2 + H_2O

H_2O-Si_2O_2

7

H2O-Si2O2 M06/maug-cc-pV(T+d)Z

O	0.036696	-0.047682	-0.015283
Si	1.704779	0.005951	-0.150547
O	1.604089	1.673930	-0.043230
Si	-0.106936	1.657826	-0.000466
O	0.012421	1.597089	2.389144
H	-0.145809	0.695502	2.684602
H	0.908318	1.823411	2.656516

trans-H(Si_2O_2)OH

7

trans-H(Si2O2)OH M06/maug-cc-pv(T+d)Z

Si	0.946572	-0.146664	-0.552350
O	2.291556	0.362430	0.177446
O	-0.105639	-1.017694	0.395686
O	-0.238637	0.999610	-0.764623
Si	-1.335147	0.110787	0.157489
H	2.246103	0.835707	1.007289
H	1.382847	-0.819148	-1.770936

cis-H(Si_2O_2)OH

7

cis-H(Si2O2)OH M06/maug-cc-pV(T+d)Z

Si	0.804521	-0.213414	-0.651403
O	2.176782	0.277485	0.044630
O	-0.199613	-1.031688	0.381763
O	-0.331064	0.986265	-0.777971
Si	-1.380241	0.167392	0.259097
H	3.019306	0.093810	-0.365651

H 1.097964 -0.914835 -1.904674

$Si_2O(OH)_2$-1

7

Si2O(OH)2-1 M06/maug-cc-pV(T+d)Z

Si	0.479197	1.787947	-1.030149
O	2.117672	1.681091	-1.052136
O	0.062192	0.447365	-0.160929
O	-0.423298	-0.613061	2.114183
Si	-0.587453	-0.915049	0.508306
H	2.563853	0.948102	-0.621139
H	-0.031372	0.210113	2.415626

$Si_2O(OH)_2$-2

7

Si2O(OH)2-2 M06/maug-cc-pV(T+d)Z

Si	1.747311	-1.084903	-0.204093
O	2.767945	-0.217915	0.744730
O	0.313567	-0.292239	0.013785
O	-0.912336	1.481753	-1.251394
Si	-1.177660	0.375058	-0.053766
H	2.456915	0.560559	1.212767
H	-1.655807	2.031782	-1.502460

$Si_2O(OH)_2$-3

7

Si2O(OH)2-3 M06-maug-cc-pV(T+d)Z

Si	1.485578	0.771230	-1.196339
O	0.573778	0.337636	0.090799
O	-1.020129	-1.488877	0.717872
O	0.507319	1.939783	-1.832178
Si	-0.307902	-0.152995	1.377873

H	0.828754	2.382867	-2.618838
H	-1.606307	-1.986234	1.290173

$Si_2O(OH)_2$-4

7

Si2O(OH)2-4 M06/maug-cc-pV(T+d)Z

Si	-1.781936	-0.242876	-0.399981
O	-1.056431	-0.967535	0.920741
O	-0.461411	-0.043674	-1.340190
O	1.635683	-0.956524	-0.137903
Si	1.188989	-0.239177	-1.538522
H	-1.625451	-1.203439	1.655501
H	0.950967	-1.137414	0.520105

c-HSiOOSiOH

7

c-HSiOOSiOH M06/maug-cc-pV(T+d)Z

Si	-1.160318	-0.775868	0.725379
O	-1.751345	0.304091	1.598636
O	-0.664716	-0.503934	-0.873830
O	0.750710	-0.845212	0.907113
Si	0.945931	-0.383067	-0.794268
H	-1.499651	-2.201289	0.796899
H	1.259870	-0.598581	1.682831

$(HSiO)_2O$

7

(HSiO)2O M06/maug-cc-pV(T+d)Z

Si	2.030701	-0.515638	-0.261825
O	2.484006	-1.537567	0.737548
O	-1.902746	-0.461897	-1.654713
O	0.530133	0.041027	-0.429923

Si -1.059168 0.213494 -0.615144

H 2.856595 0.197974 -1.232676

H -1.498950 1.179757 0.388202

OSiHOSiOH-1

7

OSiHOSiOH-1 M06/maug-cc-pV(T+d)Z

Si 2.172805 -0.148391 -0.401559

O 2.229062 1.371333 0.221212

O -1.558896 -1.293302 1.164688

O 0.529847 -0.338490 -0.401136

Si -1.004549 -0.619233 -0.058897

H 3.090799 1.773785 0.341451

H -1.800888 -0.132431 -1.186728

OSiHOSiOH-2

7

OSiHOSiOH-2 M06/maug-cc-pV(T+d)Z

Si 1.847798 0.393796 -0.986307

O 2.508942 1.108001 0.324298

O -1.370568 -1.929952 0.371637

O 0.277995 0.140589 -0.470036

Si -1.129281 -0.535708 -0.134663

H 2.023684 1.252321 1.139887

H -2.165846 0.464503 -0.395287

c-HSiSiO$_2$(OH)

7

c-HSiSiO2(OH) M06/maug-cc-pV(T+d)Z

Si -0.255677 0.672651 0.203922

O -0.341412 0.851732 1.777593

O -1.457310 0.525250 -0.768448

O	0.995722	0.719008	-0.718791
Si	-0.203531	0.600680	-2.234938
H	0.452327	0.988661	2.298422
H	-0.078830	-0.914423	-2.305709

c-HSiO$_2$SiOH

7

c-HSiO2SiOH M06/maug-cc-pV(T+d)Z

Si	0.857941	0.005255	-0.010874
O	0.915554	1.646270	-0.048639
O	-0.675066	-0.203954	1.216348
O	-0.660156	-0.265318	-1.249686
Si	-1.576725	-0.496028	-0.015907
H	0.138474	2.206836	-0.055392
H	-2.950983	-0.991562	-0.010619

HSiOSiOOH

7

HSiOSiOOH M06/maug-cc-pV(T+d)Z

Si	0.452740	0.318498	0.323750
O	0.958046	1.560203	-0.354143
O	1.105679	-0.415436	1.598602
O	-0.817030	-0.554956	-0.057265
Si	-2.013642	-1.549746	-0.618776
H	-3.139169	-0.924704	0.205169
H	1.899925	-0.011669	1.953104

HSiOOSiOH

7

HSiOOSiOH M06/maug-cc-pV(T+d)Z

Si	-1.426186	-0.848307	0.152733
O	-1.067748	-0.851848	1.621426

O	-0.332390	-0.752758	-1.009508
O	1.617533	-0.664781	0.693888
Si	1.372848	-0.645036	-0.908587
H	-2.770533	-0.929750	-0.414765
H	0.870688	-0.725099	1.321362

$H_2Si_2O_3$

7

H2Si2O3 M06/maug-cc-pV(T+d)Z

Si	0.693621	-0.003536	-0.000025
O	2.188998	-0.008699	0.000020
O	-0.463493	-1.166466	-0.000013
O	-0.456009	1.166737	-0.000010
Si	-1.666374	0.003949	0.000001
H	-2.469528	0.006519	1.224288
H	-2.469564	0.006522	-1.224261

TS21

7

TS21 M06/maug-cc-pV(T+d)Z

O	-0.015171	-0.003616	0.025072
Si	1.669084	0.011207	-0.004339
O	1.581764	1.694180	-0.000759
Si	-0.098472	1.665931	-0.016756
O	-0.564003	2.285031	1.633252
H	0.101579	2.779658	2.134206
H	-1.038458	2.853436	0.599128

TS22

7

TS22 M06/maug-cc-pV(T+d)Z

O	0.005764	-0.085007	0.087291
Si	1.693810	0.009335	-0.114478
O	1.593757	1.653751	0.076342
Si	-0.108564	1.834096	-0.006912
O	-0.545773	1.357815	1.757110
H	0.066224	1.597005	2.458790
H	-0.311588	0.319130	1.215273

TS23

7

TS23 M06/maug-cc-pV(T+d)Z

O	0.157017	0.594619	0.279470
Si	1.402957	-0.405772	-0.313127
O	0.805891	-0.185493	-1.843528
Si	-0.368840	1.000054	-1.513312
O	-1.698668	-0.134505	-0.791176
H	-2.586779	0.226419	-0.736129
H	-0.969719	0.076409	0.113003

TS24

7

TS24 M06/maug-cc-pV(T+d)Z

Si	0.109537	0.006469	1.139262
O	0.614024	1.422539	1.726549
O	-1.064456	0.189210	-0.011847
O	1.108751	-0.672120	0.000740
Si	-0.078818	-0.507471	-1.190626
H	1.527642	1.590559	1.946020
H	-0.265630	-0.899617	2.224042

TS25

7

TS25 M06/maug-cc-pV(T+d)Z

Si	2.017226	-0.579543	-0.250574
O	2.458432	-0.696333	1.326111
O	0.423843	-0.161972	-0.141246
O	-1.116432	1.871644	0.084581
Si	-1.158925	0.268038	-0.245964
H	1.829240	-0.501510	2.024773
H	-1.094457	2.668226	-0.438803

TS26

7

TS26 M06/maug-cc-pV(T+d)Z

Si	-0.750083	1.909748	-0.450976
O	0.620859	2.805185	-0.589072
O	-0.139393	0.407112	-0.139207
O	0.184407	-1.124858	1.887092
Si	0.191756	-1.154908	0.249611
H	1.479383	2.389840	-0.478134
H	0.851821	-1.043118	2.562686

TS27

7

TS27 M06/maug-cc-pV(T+d)Z

Si	-0.476717	-0.281285	1.974236
O	-0.520242	-0.272386	0.330764
O	1.094825	-0.354416	-1.574111
O	-0.581615	1.310163	2.346424
Si	-0.535634	-0.369674	-1.301002
H	0.056167	1.983974	2.566101
H	1.374895	-0.415605	-2.488483

TS28

7

TS28 M06/maug-cc-pV(T+d)Z

Si	-0.650887	-0.926185	1.708508
O	-0.574578	-0.331549	0.176800
O	1.157130	0.167602	-1.566493
O	-0.687067	0.430721	2.623827
Si	-0.483865	0.133122	-1.388419
H	-1.379485	0.949100	3.024629
H	1.486493	0.415820	-2.431553

TS29

7

TS29 M06/maug-cc-pV(T+d)Z

Si	-1.839344	-0.494189	-0.579958
O	-1.311760	-1.118318	0.867135
O	-0.453571	0.060126	-1.267348
O	1.552685	-1.090416	-0.123372
Si	1.215414	0.033950	-1.270322
H	-1.131791	-0.685276	1.700646
H	0.818777	-1.496516	0.352969

TS30

7

TS30 M06/maug-cc-pV(T+d)Z

Si	0.004445	-0.978282	0.448004
O	0.209975	-0.667753	2.025297
O	-1.046112	0.152434	-0.194160
O	1.258297	-0.072501	-0.675033
Si	-0.019663	0.863815	-1.307002
H	-0.079865	0.175675	2.379430
H	1.064564	-1.358927	-0.695966

TS31

7

TS31 M06/maug-cc-pV(T+d)Z

Si	-0.916130	-0.546746	0.326934
O	-1.839790	0.722798	0.755551
O	-0.188150	-0.101287	-1.103277
O	0.832290	-0.393154	1.004009
Si	1.396642	-0.076192	-0.586089
H	-2.494087	0.587903	1.440911
H	0.128794	-1.475101	1.129350

TS32

7

TS32 M06/maug-cc-pV(T+d)Z

Si	-0.980199	-0.907874	0.686128
O	-1.039093	0.146087	1.820022
O	-0.652113	-0.496592	-0.897666
O	0.840736	-0.917140	0.888818
Si	0.998452	-0.447065	-0.786732
H	-1.668567	-2.196237	0.721467
H	0.381264	-0.185039	1.610721

TS33

7

TS33 M06/maug-cc-pV(T+d)Z

Si	-1.617706	-0.784331	-0.076117
O	-1.881184	-0.231248	1.298495
O	-0.279921	-0.582785	-0.943200
O	1.517842	-1.115269	0.779014
Si	1.347377	-0.325278	-0.637515
H	-2.561540	-1.565780	-0.877026
H	1.593343	-0.847469	1.692198

TS34

7

TS34 M06/maug-cc-pV(T+d)Z

Si	-1.380890	-0.354314	-0.510745
O	-1.433157	-0.625714	1.025997
O	0.018309	-0.383326	-1.300242
O	1.519715	-1.034143	0.724363
Si	1.631541	-0.706122	-0.866289
H	-2.720554	-0.436554	0.239745
H	0.689367	-1.016086	1.220632

TS35

7

TS35 M06/maug-cc-pV(T+d)Z

Si	0.142277	0.790866	0.442911
O	1.721290	0.756925	0.315426
O	-0.680199	0.469579	1.734473
O	-0.924894	1.252834	-0.605152
Si	-1.997460	0.019370	0.424525
H	2.147604	0.957859	-0.519485
H	-0.908597	-1.036432	-0.055479

TS36

7

TS36 M06/maug-cc-pV(T+d)Z

Si	-0.251759	0.228546	0.387620
O	-0.356203	1.069692	1.751565
O	-1.388850	0.387424	-0.742417
O	0.968373	0.399799	-0.655739
Si	-0.166193	0.646087	-1.965671
H	0.433177	1.361337	2.210539
H	-0.127255	-0.649325	-2.733847

TS37

7

TS37 M06/maug-cc-pV(T+d)Z

Si	0.764951	-0.151862	-0.124933
O	1.039262	1.373362	-0.414059
O	-0.209575	-0.118506	1.249045
O	-0.607843	-0.481006	-1.044130
Si	-1.408647	0.316362	0.178568
H	-0.389916	1.696932	-0.217709
H	-2.819202	0.008585	0.470901

TS38

7

TS38 M06/maug-cc-pV(T+d)Z

Si	0.011721	0.737692	0.183252
O	-0.422651	0.818920	1.717809
O	-1.235006	0.543166	-0.777647
O	1.370098	0.845801	-0.476839
Si	-0.653586	0.497700	-2.404183
H	0.255657	0.963313	2.379443
H	-0.214944	-0.963030	-2.369786

TS39

7

TS39

Si	0.468306	0.173203	-0.523980
O	1.382210	0.488459	-1.666064
O	0.671920	0.175500	1.112257
O	-1.068962	-0.349833	-0.434495
Si	-1.182295	-0.509360	1.239013
H	-2.040705	0.616558	1.697105
H	0.314336	-0.929096	1.676454

TS40

7

TS40

Si	0.858692	0.005567	-0.030415
O	0.917429	1.646622	-0.038828
O	-0.683843	-0.227588	1.240537
O	-0.656113	-0.238625	-1.227985
Si	-1.577828	-0.494868	0.001714
H	0.140773	2.205784	-0.090420
H	-2.950070	-0.995393	-0.029372

TS41

7

TS41 M06/maug-cc-pV(T+d)Z

Si	-0.084503	-0.592092	2.050607
O	0.163946	0.940550	2.524776
O	-1.311515	-0.225381	-1.995407
O	0.086797	-0.474884	0.399046
Si	-0.058741	-0.426915	-1.190064
H	0.856974	1.505277	2.853931
H	1.276722	-0.634394	-1.754139

TS42

7

TS42 M06/maug-cc-pV(T+d)Z

Si	-0.169219	-0.631527	2.100251
O	-0.231802	0.961350	2.426319
O	-1.179778	-0.204618	-1.944542
O	0.132821	-0.634524	0.463748
Si	0.056567	-0.422341	-1.117258
H	-0.900894	1.630855	2.541393
H	1.420074	-0.505665	-1.643512

TS43

7

TS43 M06/maug-cc-pV(T+d)Z

Si	1.971219	-0.745324	0.034804
O	2.881069	0.252636	0.687039
O	-2.117279	-1.124335	1.152900
O	0.362462	-0.660983	-0.036656
Si	-1.214478	-0.350584	0.163765
H	2.363153	-1.960516	-0.675608
H	-2.728215	-0.124975	0.141228

TS44

7

TS44 M06/maug-cc-pV(T+d)Z

Si	0.344986	-0.321410	-2.147585
O	1.069605	1.151195	-2.053077
O	0.482955	-1.324252	2.022578
O	-0.124782	-0.454005	-0.564281
Si	-0.419578	-0.571718	1.009079
H	1.455964	1.508703	-2.854199
H	-0.891321	-0.806591	2.448673

TS45

7

TS45 M06/maug-cc-pV(T+d)Z

Si	0.999930	-0.168480	-0.293110
O	2.498855	0.000760	-0.000020
O	-0.161667	-1.052199	0.456964
O	-0.134056	0.897316	-0.814066
Si	-1.373789	0.017376	-0.059542
H	2.218503	-0.776101	-1.195122
H	1.454129	-1.079307	-1.676828

TS46

7

TS46 M06/maug-cc-pV(T+d)Z

Si	0.462985	0.105533	0.004808
O	1.886876	0.561674	0.022561
O	-0.626747	-0.319627	1.159446
O	-0.626672	-0.230036	-1.178935
Si	-1.770195	-0.669948	-0.024891
H	-2.662655	0.849211	0.033168
H	-3.200262	-0.297788	-0.010776

TS47

7

TS47 M06/maug-cc-pV(T+d)Z

Si	-1.170388	-0.458783	0.369260
O	-2.374883	0.244922	0.917611
O	-0.410420	-0.304482	-1.095048
O	0.457805	-0.636172	1.045084
Si	1.155002	-0.374397	-0.533020
H	-1.130046	-2.083214	0.662899
H	-0.204432	-1.724593	0.997803

TS48

7

TS48 M6/maug-cc-pV(T+d)Z

Si	0.354126	-0.069043	-0.021748
O	1.658084	-0.002877	-0.752959
O	-0.050475	-0.007312	1.559150
O	-1.191722	-0.191504	-0.525424
Si	-2.021841	0.052241	0.928354
H	-0.772455	-0.989998	1.790553
H	-1.664048	-1.472125	1.680593
